# A diffusion tensor imaging white matter atlas of the domestic canine brain

**DOI:** 10.1162/imag_a_00276

**Published:** 2024-08-30

**Authors:** Fiona M. Inglis, Paul A. Taylor, Erica F. Andrews, Raluca Pascalau, Henning U. Voss, Daniel R. Glen, Philippa J. Johnson

**Affiliations:** Cornell College of Veterinary Medicine, Department of Clinical Sciences, Cornell University, Ithaca, NY, United States; Scientific and Statistical Computing Core, National Institute of Mental Health, Bethesda, MD, United States; Faculty of Medicine, “Iuliu Hatieganu” University of Medicine and Pharmacy, Cluj-Napoca, Romania; Cornell Magnetic Resonance Imaging Facility, College of Human Ecology, Cornell University, Cornell, Ithaca, NY, United States

**Keywords:** DTI, dog, fasciculi, tractography, template, tract, subcortical

## Abstract

There is increasing reliance on magnetic resonance imaging (MRI) techniques in both research and clinical settings. However, few standardized methods exist to permit comparative studies of brain pathology and function. To help facilitate these studies, we have created a detailed, MRI-based white matter atlas of the canine brain using diffusion tensor imaging. This technique, which relies on the movement properties of water, permits the creation of a three-dimensional diffusivity map of white matter brain regions that can be used to predict major axonal tracts. To generate an atlas of white matter tracts, thirty neurologically and clinically normal dogs underwent MRI imaging under anesthesia. High-resolution, three-dimensional T1-weighted sequences were collected and averaged to create a population average template. Diffusion-weighted imaging sequences were collected and used to generate diffusivity maps, which were then registered to the T1-weighted template. Using these diffusivity maps, individual white matter tracts—including association, projection, commissural, brainstem, olfactory, and cerebellar tracts—were identified with reference to previous canine brain atlas sources. To enable the use of this atlas, we created downloadable overlay files for each white matter tract identified using manual segmentation software. In addition, using diffusion tensor imaging tractography, we created tract files to delineate major projection pathways. This comprehensive white matter atlas serves as a standard reference to aid in the interpretation of quantitative changes in brain structure and function in clinical and research settings.

## 1 Introduction

The domestic canine, *canis familaris*, has gained increasing recognition as a model for human disease. Many diseases with human homology occur spontaneously in canines, with similar underlying pathology and progression as the human condition. Canines are uniquely suited to studies of disease within the nervous system. For example, unlike rodents which are common subjects for neuroscience research, the canine brain is gyrencephalic, with similar cortical divisions to the human brain. Dogs display similar executive cognitive functions to humans (Bunford et al., 2017), and are naturally prone to a number of conditions that affect humans such as age-related changes in cognitive function (Dewey et al., 2019, 2020; Vite & Head, 2014), anxiety disorders (Blackwell et al., 2013; Dreschel, 2010), epilepsy (Löscher, 2022), glioma (Hicks et al., 2017; Hubbard et al., 2018; Leblanc et al., 2016), neuromuscular disorders (Nghiem & Kornegay, 2019), neuropathies (Correard et al., 2019), retinopathies (Petersen-Jones & Komáromy, 2015), and metabolic diseases (Gurda & Vite, 2019). Widespread acceptance of dogs as companion animals ensures a canine population with similar environmental exposures to humans in which disease progression is often charted. Furthermore, in contrast to inbred strains of lab animals, domestic canines exhibit breed-related differences in disease prevalence that have provided insight into genetic predispositions for developing various neurological disorders (Dickinson, 2020; Dickinson & Bannasch, 2020; Ekenstedt & Oberbauer, 2013; Flegel et al., 2021; Garafalo et al., 2020; Hülsmeyer et al., 2015; Nardone et al., 2016; Wu et al., 2018).

In neuroscience research, there is a need for methods to map neurological function, and to measure the effects of diseases on both function and structure. Magnetic resonance imaging (MRI) represents a non-invasive methodology that permits study of the brain’s anatomic detail and can provide information regarding structure-function relationships. In MRI diffusion tensor imaging (DTI), patterns of diffusion of water molecules can be used to assign a value for isotropy for different tissue types. Within brain tissues, diffusion of water is constrained by cellular architecture, and the amount of constrained diffusion, or fractional anisotropy (FA), can be attributed to specific tissues. In white matter, diffusion is constrained in a bidirectional manner, and high values for FA can be used to delineate anatomically defined functional white matter tracts that may not be discriminated histologically (Bazin et al., 2011; Le Bihan, 2003; Mori & Zhang, 2006). Accordingly, white matter atlases employing DTI provide a standardized reference that can be used to investigate structure-function relationships, such as age-related (Calabrese et al., 2015; Mukherjee & McKinstry, 2006; Qiu et al., 2008; F. Zhang et al., 2018) and disease-related changes in brain connectivity (Alexander et al., 2007; Bonilha et al., 2013; Freund et al., 2010; Johnson, Barry, et al., 2019). DTI can also be used to establish models of diffusion tensors that create deterministic or probabilistic orientation paths of white matter (Mori et al., 2009).

White matter atlases have been established for several species used in research, including non-human primates (Calabrese et al., 2015; Liu et al., 2020; Oishi et al., 2011; Zakszewski et al., 2014), rodents (Figini et al., 2015; Jiang & Johnson, 2011; Yon et al., 2020), cats (Jacqmot et al., 2017; Johnson, Pascalau, et al., 2020; Stolzberg et al., 2017), sheep (Nitzsche et al., 2015; Pieri et al., 2019), and horses (Boucher et al., 2020; Johnson, Janvier, et al., 2019), permitting comparative studies of white matter tracts across species and with human subjects (Catani & Thiebaut de Schotten, 2008; Mori et al., 2008, 2011). DTI has also been employed to delineate specific tracts within the canine brain (Anaya García et al., 2015; Jacqmot et al., 2013, 2017) and to detect alterations in canine brain diffusivity in the aging brain (Barry et al., 2021), epilepsy (Hamamoto et al., 2017), and glaucoma (Graham et al., 2021). Recently, DTI has been used to identify olfactory pathways not previously described (Andrews et al., 2022). Beyond clinical applications, these studies pose a useful corollary to the nascent field of canine functional MRI (fMRI) in comparative studies of function and evolution (Andics & Miklósi, 2018). While comparative studies of cognition employing dogs cannot replace those performed in non-human primates, whose cognitive and executive functions are closely aligned with humans, dogs as a species are well-suited to studies of cognition, since they are able to recognize and interpret human verbal and non-verbal language, and respond to human commands (Andics & Miklósi, 2018; Bunford et al., 2017). Studies employing fMRI have shown convergence in human and canine neural processing in temporo-occipital brain regions when presented with animate versus inanimate objects (Boch et al., 2023; Bunford et al., 2020), and activation of canine olfactory pathways associated with species-specific recognition (Boch et al., 2023). While these techniques provide complementary mechanisms for functional comparisons of distinct grey matter areas and white matter pathways, to date, however, canine white matter has not been comprehensively parcellated in an atlas form.

Here, we present a DTI white matter atlas for the mesaticephalic canine brain, created using diffusion-weighted imaging and 3-dimensional T1-weighted data from 30 neurologically normal dogs. In this atlas, we provide a series of white matter segmentation maps and tract priors available for download in common neuroimaging informatics technology initiative (NIfTI) format (Cox et al., 2004). This comprehensive white matter atlas establishes a reference standard that aids standardization of data processing and interpretation of MRI images in canine brain research and may be used to facilitate studies of functional alterations in connectivity with respect to canine neurological disease.

## 2 Materials and Methods

### 2.1 Study population

For creation of population average templates, we recruited 30 neurologically normal dogs from research populations housed at Cornell College of Veterinary Medicine. Since cranial conformation in brachycephalic dogs has been demonstrated to result in frontal shortening of the brain (Johnson, Luh, et al., 2020), only mesaticephalic and dolichocephalic dogs were included. The final cohort was composed of 10 beagles and 20 mixed breed dogs that had a median weight of 13 kg (IQR 12.75) and had either a mesaticephalic (n = 25) or dolichocephalic (n = 5) cranial conformation. This group was composed of eight males and 22 females, with a median age of 5.5 years (IQR 7.5). All animals were clinically healthy and neurologically normal and underwent MRI for research purposes only.

### 2.2 Ethics statement

This research was approved by Cornell University’s institutional animal care and use committee (IACUC protocol number: 2015-0115).

### 2.3 MRI examination

All dogs underwent physical and neurological examinations to ensure that they were clinically healthy, neurologically normal and had an American Society of Anesthesiologists (ASA) score of I. They were premedicated, induced, and maintained under anesthesia by a board-certified veterinary anesthesiologist as previously described (Johnson, Luh, et al., 2020). MRI was performed using a 3.0T General Electric (GE) Discovery MR750 whole-body scanning unit. Subjects were imaged in dorsal recumbency using a 16-channel radio-frequency coil (Johnson, Luh, et al., 2020). Both T1-weighted 3D inversion recovery fast spoiled gradient echo (BRAVO, isotropic voxels 0.5 mm^3^, TE = 3.6 ms, TR = 8.4 ms, TI = 450 ms, excitations = 3, flip angle 12°, acquisition matrix size 256 x 256) and diffusion tensor imaging (TR = 7000 ms, TE = 89.6 ms, flip angle = 90°, isotropic voxels 1.5 mm^3^, 60 gradient directions, b = 800 s/mm^2^ and a single unweighted (b = 0) diffusion image) sequences were performed on each subject.

### 2.4 Data processing

DW images were corrected for phase distortion (Andersson et al., 2003; Smith et al., 2004), eddy current distortion, motion correction (Andersson & Sotiropoulos, 2016), Gibbs artifact (Kellner et al., 2016), and noise (Veraart et al., 2016) using the FSL (https://fsl.fmrib.ox.ac.uk/) (Smith et al., 2004) and MRTrix (https://www.mrtrix.org) (Tournier et al., 2019) software packages. Diffusion tensors were modeled using the three principal eigenvalues with FSL’s *dtifit* from the FSL diffusion toolbox (Behrens et al., 2003, 2007). Tensor maps were calculated for fractional anisotropy (FA) using the following equation (Basser & Jones, 2002; Beaulieu, 2002).

$$\sqrt{\frac{{\left(\lambda_{1}-\lambda_{2}\right)}^{2}+{\left(\lambda_{2}-\lambda_{3}\right)}^{2}{\left(\lambda_{1}-\lambda_{3}\right)}^{2}}{2(\lambda_{1}^{2}+\lambda_{2}^{2}+\lambda_{3}^{2})}}.$$

In addition, maps for mean diffusivity (MD; $(\lambda_{1}+\lambda_{2}+$ $\lambda_{3})/3$), radial diffusivity (RD; $(\lambda_{2}+\lambda_{3})/2$), and axial diffusivity (AD; λ_1_) were generated for all subjects. All maps were visually inspected for quality assurance between each stage of registration, orientation, and preprocessing.

### 2.5 Population template creation

#### 2.5.1 T1-weighted population average template

A T1-weighted template was created as described previously (Johnson, Luh, et al., 2020). This template underwent quality assurance testing and the success of registration after aligned, linear, and non-linear registration was performed with external datasets was evaluated as previously described (Johnson, Luh, et al., 2020). This template was used for manual white matter mask delineation and figure creation.

#### 2.5.2 DTI population average template

Using MRTrix tools (Tournier et al., 2019), preprocessed diffusion data and brain masks for all subjects were used to generate fiber orientation distributions (FODs). The resulting MRtrix population template was then used to generate an unbiased average FOD data set for the canine subjects employed in this study. To generate an average diffusion data set, the average FOD was subsequently converted back into diffusion data using the fod2dwi tool in MRtrix (Tournier et al., 2019; Wang et al., 2007). The average population data were subject to deterministic modeling using Diffusion Toolkit. Using the template diffusion tractogram, virtual dissection was performed in TrackVis (Wang et al., 2007). This method produced a population average diffusion data set representing our canine sample that could be used for tractography.

### 2.6 Manual white matter segmentation

Anatomic regions of white matter were manually segmented using ITK-Snap (Yushkevich et al., 2006) (www.itksnap.org), using sagittal, transverse, and dorsal planes to ensure accurate reconstruction of three-dimensional white matter structures. Segmentations were performed on the T1-weighted population average template, and fiber orientation was visualized by overlying color-coded red-blue-green FA maps to help define borders between adjacent white matter structures. White matter segmentations were made with reference to existing white matter atlases created for other species (Calabrese et al., 2015; Mori et al., 2011; Oishi et al., 2011; Zakszewski et al., 2014) and canine histological and MRI atlases (Fletcher & Saveraid, 2009; Nitzsche et al., 2019; Singer, 1962). Segmentations divided the white matter according to anatomic structures visible on the population average templates, such as within specific gyri or defined subcortical structures.

### 2.7 Deterministic tractography dissection

Tractography dissections of the population average diffusion dataset tractogram were performed using TrackVis software (Johnson, Pascalau, et al., 2020). The major projection, association, commissural, and cerebellar fasciculi were dissected using both human and animal anatomic references (Catani & Thiebaut de Schotten, 2008; Jacqmot et al., 2013, 2017; Johnson, Pascalau, et al., 2020). Descriptions of how each were dissected are included under each tract title in the results section, and a guide to region of interest placement is included in Supplementary Materials.

### 2.8 Template and atlas collection processing and formatting

Several aspects of the template and atlas were processed using AFNI (Cox, 1996) to ensure maximal usability and consistency among the various parts of the collection. For example, we ensured that all expected parts of the NIFTI header (Cox et al., 2004) were present and correct, including srow values and appropriate qform_code and sform_code values of 5. The dataset datatypes are short in order to have efficient disk space usage for these files, which should also help create efficiently-sized files for downstream processing by default. The dataset orientation was set to be “RAI,” for convenience of interpretation and simpler mapping between data matrix and physical coordinate location across general software and scripting. The coordinate origin (x, y, z) = (0, 0, 0) itself was placed in a convenient and meaningful anatomical location within the brain (at the anterior commissure), facilitating interpretations of reported results, mapping to other templates and spaces (especially across species), and likely initial overlap with acquired datasets that researchers will look to align with the template. The final voxel size (0.5 mm isotropic) is also a convenient number for reporting coordinates. The template dataset, atlases, network maps, and masks were all verified for consistency of coverage and overlap both visually and computationally.

**Fig. 1.**
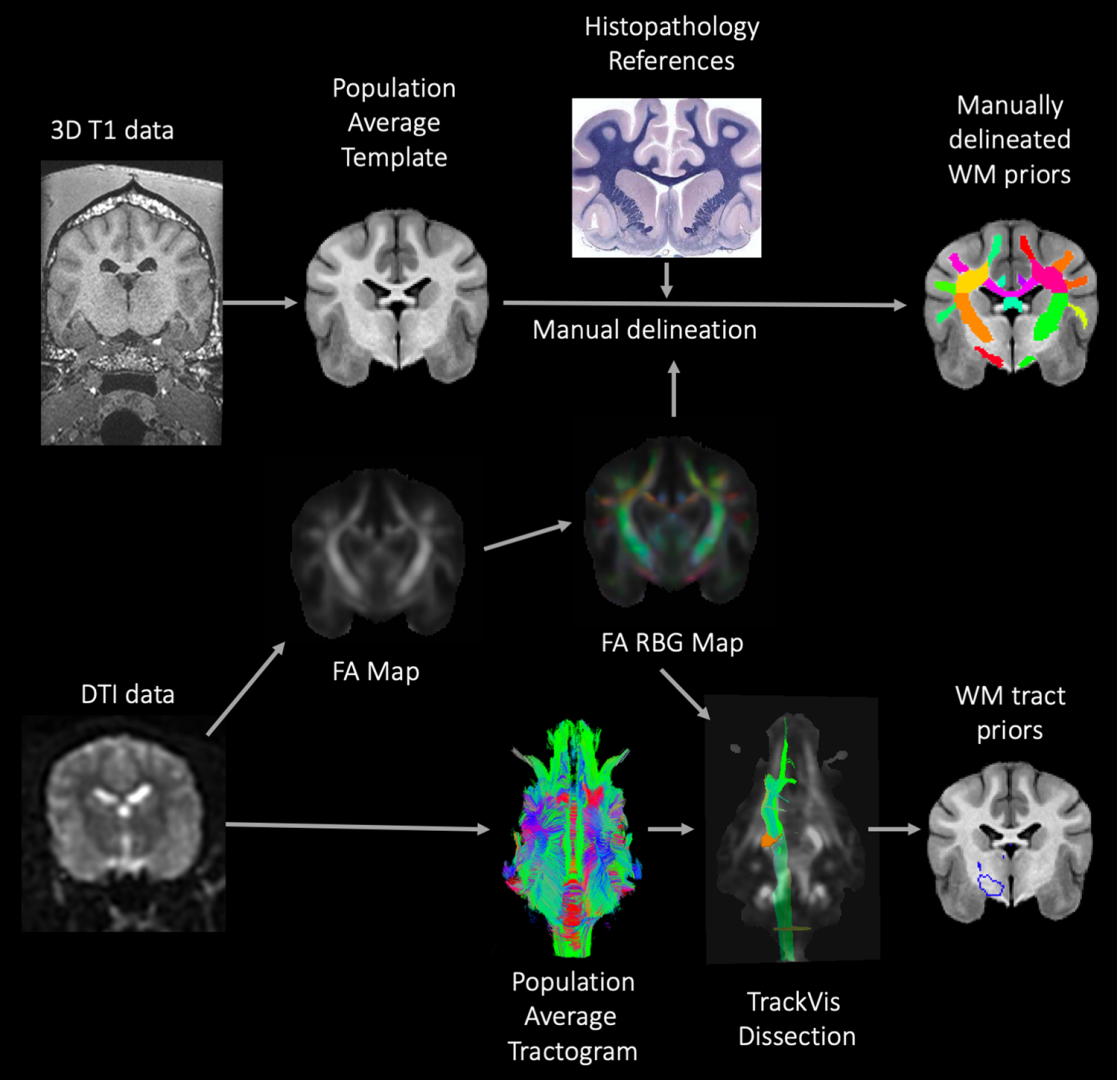
Provides an overview of the methods used to create the manually delineated white matter priors and white matter tract priors that make up the final white matter (WM) atlas. A population average template was created from high-resolution isovolumetric 3-dimensional (3D) T1W data. The WM priors were created by manually delineating the different regions of the WM using anatomical and histopathological references and evaluation of the fractional anisotropy (FA) red blue green (RBG) color map. In order to create representative population average WM tract priors, the raw diffusion tensor imaging (DTI) data were corrected for distortion, motion, artifact, and noise using FSL and MRtrix software. FSL DTIfit was used to model diffusion tensors and create FA maps. A diffusion-weighted population average template was created using MRtrix. Diffusion toolkit was used to deterministically model the population average DTI data to create a tractogram. This population average tractogram was dissected using TrackVis software with reference to the FA RBG color map. Individual tracts were saved as masks and registered to the population average T1W template.

For all relevant atlas files, label tables were created with AFNI’s @MakeLabelTable to insert that information directly into the file header. This is convenient within AFNI’s widely used visualization software for displaying atlas regions within the GUI, as well as interactively clicking around and seeing lists ROIs located at and near the current click location (called “Where am I?” functionality). Programmatically, users can also use the label names to select regions conveniently within commands, which is less likely to result in scripting errors and is easier for others to correctly interpret. Plain text label files were also created for general reference, and these are distributed within the data package. (A schematic of the methods used is documented in Fig. 1.)

### 2.9 White matter atlas

FSLmaths and FSLstats were used to document the volume and mean FA and MD values for each of the white matter anatomic priors. The final atlas is available, open source at: https://doi.org.10.7298/2w4q-8j27 and https://hdl.handle.net/1813/113846

## 3 Results

### 3.1 White matter regions—manual segmentation

#### 3.1.1 Gyral white matter

White matter within each gyrus represents the terminal extensions of white matter within the corona radiata, a large central white matter region that fans out as it extends dorsally (see below). Here, we have segmented gyral white matter as follows. Each separate gyral parcellation was considered to begin as the corona radiata narrowed to enter the gyrus. For each, the left and right gyrus was segmented separately. Segmentations were performed with reference to previous studies of white matter segmentation in canines (Fletcher & Saveraid, 2009; Nitzsche et al., 2019) and pre-existing canine histological (Adrianov, 2010; Singer, 1962) and MRI atlases (Czeibert et al., 2019).

*Cingulum:* This association tract runs in a predominantly rostral/caudal direction dorsal to the corpus callosum (Schachter & Singer, 1962) and was dissected with reference to manual segmentation of the rhesus macaque brain (Zakszewski et al., 2014). At the caudal aspect of the splenium of the corpus callosum, the cingulum courses dorsoventrally, giving rise to the perihippocampal cingulum. This limbic structure contains both long- and short-range cortico-cortical fibers, as well as axons arising from the amygdala, thalamus, and brainstem nuclei, and provides afferent innervation to the hippocampus and parahippocampal gyrus (Vogt, 2019). While short and long association fibers of the cingulum defasciculate towards individual gyri, the cingulum bundle dissection was limited to the white matter running rostrocaudally.

*Splenial gyrus:* This small, predominantly rostrocaudally orientated gyrus lies dorsal to the cingulum on the midline surface of the falx cerebri, adjacent to the marginal gyrus. At its caudal end, the splenial gyrus courses dorsoventrally, caudal to the perihippocampal cingulum (Czeibert, Baksa, et al., 2019).

*Marginal, Ectomarginal, Suprasylvian, Ectosylvian and Sylvian Gyri:* These gyri, listed from medial to lateral, have a predominantly rostral/caudal orientation and comprise the majority of the surface of the parietal, sensorimotor, occipital, and temporal regions of the brain (Czeibert, Andics, et al., 2019; Johnson, Luh, et al., 2020).

*Rostral and caudal composite gyri:* These ventrolaterally located gyri adjoin the sylvian, ectosylvian, and suprasylvian gyri at their rostral and caudal extent. The borders of the rostral and composite gyri are drawn at the acute angle where they converge with the silvian, ectosylvian, and suprasylvian gyri (Czeibert, Andics, et al., 2019).

*Precruciate and post-cruciate gyri:* These gyri lie rostral and caudal to the cruciate sulcus respectively and have a predominantly medial/lateral orientation. The border between these is drawn at the most lateral aspect of each gyrus.

*Frontal, Rectus* and *Proreus gyri* were delineated according to the study by Czeibert and colleagues (Czeibert, Andics, et al., 2019) and are as follows:

*Frontal gyrus:* This gyrus runs in a rostral/caudal orientation within the frontal region of the brain, and occupies a mediodorsal position, rostral to the cruciate sulcus and its associated gyri.

*Rectus gyrus:* This white matter region lies within the medial aspect of the frontal region, ventral to the frontal gyrus, and runs rostrocaudally.

*Proreus:* This gyrus is situated within the frontal region and lies lateral to the rectus gyrus. It runs in a rostrocaudal orientation dorsal to the olfactory lobes.

#### 3.1.2 Central white matter

*Corona radiata:* The corona radiata represents the white matter of the cortical gyri and extends ventrally to the internal capsule. Fibers within the corona radiata form a fan-like structure dorsal to the internal capsule. Tracts within these regions include corticothalamic and thalamocortical pathways, cortical projections to subcortical extrapyramidal structures, and corticofugal pathways including corticopontine, corticobulbar, and corticospinal pathways (de Lahunta et al., 2021b). The corona radiata also conveys long- and short-range cortico-cortical association fibers. Extensions of the corona radiata were subdivided in this atlas as they enter individual gyri and segmented as above. Gyri of the pariental, temporal, and occipital lobes were segmented from medial to lateral as marginal, ectomarginal, suprasylvian, ectosylvian, and sylvian white matter (Czeibert, Andics, et al., 2019; Datta et al., 2012; Nitzsche et al., 2019). Gyri within prefrontal and frontal areas were segmented as rostral suprasylvian, post-cruciate, precruciate, frontal, prorean, and rectus gyri. At their rostral and caudal margins, sylvian, ectosylvian and suprasylvian gyri are connected via the rostral and caudal composite gyri respectively.

*Internal capsule:* This white matter structure represents a continuation of the corona radiata ventrally. The margin between the corona radiata and internal capsule was arbitrarily defined in this atlas on the transverse plane as the approximate dorsal margin of the caudate. This definition is in line with previous histological and MRI references (Czeibert, Andics, et al., 2019; Singer, 1962). At the caudoventral extent, the internal capsule continues as the cerebral peduncle. The internal capsule contains corticothalamic and thalamocortical fibers, and contains the long corticofugal fibers including the corticospinal, corticobulbar, and corticopontine tracts.

*Temporal Horn:* White matter within the temporal horn is distinguished from the internal capsule in this atlas, since information within the temporal lobe represents predominantly limbic information and auditory processing in the dog, and pathways within the temporal horn are required for auditory recognition and spatial memory (Kowalska, 2000). Tracts within this region enter the temporal lobe, occipital cortex (Johnson, Luh, et al., 2020), and olfactory structures (Andrews et al., 2022). The rostral border with the internal capsule was arbitrarily defined as the approximate rostral margin of the lateral geniculate nucleus, where the internal capsule is at its most lateral position.

*Cerebral peduncle:* The cerebral peduncle continues ventrally and caudally from the internal capsule, conveying ascending sensory and descending motor tracts between the cerebrum and pons. These tracts contain the corticopontine, corticobulbar, and corticospinal tracts. The cerebral peduncle represents an anatomical eminence visible on the ventral surface of the canine brain, and was delineated as such.

*Perithalamic Tract:* This prominent white matter tract begins at the optic chiasm and conveys visual afferent information from the optic nerve. In the dog, an estimated 75% of fibers cross at the optic chiasm (de Lahunta et al., 2021c) and run contralaterally lateral to the thalamus to terminate at the lateral geniculate nucleus. The right and left optic tracts were segmented.

*Corpus Callosum (CC):* This prominent midline white matter tract contains commissural fibers connecting analogous cortical regions in both hemispheres. The orientation of this structure within the rostral two thirds is rostral/caudal, and projects in with similar rostro-caudal topography to the cortex. While this represents a continuous white matter structure, we subdivided the corpus callosum into the following divisions: genu, body, splenium, and tapetum in alignment with previous studies in other species. The borders of each were drawn arbitrarily but correspond approximately to divisions in humans (Catani & Thiebaut de Schotten, 2008; Mori et al., 2008, 2011; Oishi et al., 2011) and non-human primates (Calabrese et al., 2015; Oishi et al., 2011; Zakszewski et al., 2014). At the caudal extremity, the corpus callosum is oriented dorsoventrally, giving rise to the tapetum which provides the temporal lobe with interhemispheric connections. The left and right tapetum were segmented separately.

*Fornix:* The rostral extremity of the fornix represents a midline structure arising at the level of the medial septum, coursing dorsally and caudally to lie directly below the corpus callosum. From there, the fornix divides and follows the lateral aspect of the hippocampus caudally and ventrally. This tract contains the septohippocampal and subiculothalamic pathways, and connects the hippocampus with several limbic regions including the amygdala, subiculum, thalamus, and mamillary bodies. This evolutionarily conserved structure is critical in formation of certain types of memory (Manns & Eichenbaum, 2006). The fornix was segmented as a single midline structure.

*Rostral Commissure:* This transverse white matter structure delimits the rostral aspect of the diencephalon and carries information from the olfactory lobes, amygdale, and pyriform cortex. This structure is prominent in T1w images, but is not visible in FA maps, likely due to the presence of substantial isotropy within the adjacent grey matter and the presence of crossing fibers within the commissure. Therefore, the rostral commissure was delineated using the T1w template rather than the FA map.

*Olfactory Peduncle:* This prominent white matter bundle contains olfactory tracts from the olfactory bulb at the rostroventral extremity of the brain. This tract is orientated in a rostral/caudal direction. Olfactory white matter was sub-divided into additional masks as a result of recent findings that indicate five distinct white matter tracts (Andrews et al., 2022).

#### 3.1.3 Cerebellar white matter

Afferents and efferents of the cerebellum can be divided into three distinct major tracts, the rostral, middle, and caudal cerebellar peduncles. These integrate information between the spinal cord, brainstem, and forebrain (de Lahunta et al., 2021a). These are parcellated according to the direction of diffusion tensors within each structure, and with reference to previous histological studies (Singer, 1962).

*Rostral Cerebellar Peduncle (RCP):* This tract conveys predominantly cerebellar efferent pathways to the mesencephalon, diencephalon, and telencephalon, and some cerebellar afferent pathways from the mesencephalon. The RCP is oriented rostrocaudally and is medial to the middle and caudal cerebellar peduncles.

*Middle Cerebellar Peduncle (MCP):* This laterally situated peduncle conveys cerebellar afferent information from the cortex via the pons. Fibers arising in the pons decussate via the transverse fibers in the ventral pons and continue as the contralateral MCP.

*Caudal Cerebellar Peduncle (CCP):* This peduncle conveys afferent information to the cerebellum from the spinal cord and vestibular system, and efferent pathways from the cerebellar nuclei to the brainstem.

*Cerebellar Medulla:* The white matter of the cerebellar hemispheres and its extensions into the overlying folia (arbor vitae) contain cerebellar afferents and axons of cerebellar Purkinje neurons that innervate cerebellar nuclei to provide critical feedback and efferent control of motion and body position (de Lahunta et al., 2021a). The cerebellar medulla was segmented as four masks, representing white matter within left and right rostral and caudal cerebellar lobes.

### 3.2 White matter tracts—deterministic tractography

#### 3.2.1 Projection tracts

*Corticospinal Tract (CST)*: The CST runs from the ventral horn of the spinal cord, through the cerebral peduncles and to form connection with the frontal and sensorimotor cortex. It was dissected by including fibers that extended between seed regions placed within the spinal cord and cerebral peduncles.

*Thalamic Radiation*: The thalamic radiation has fibers running between the thalamus and cerebral cortex. It lies in close association with the CST; however, it extends within the corona radiata to form a more extensive cortical connectivity involving frontal, sensorimotor, parietal, and occipital regions. It was dissected by using a seed region within the thalamus and excluding pathways that extended into the brainstem.

*Fornix:* The fornix extends between the mammillary bodies and hippocampus. It was dissected by using including fibers that ran between regions within the body and crura of the fornix.

*Optic tract and radiation:* The optic pathway was dissected as previously described (Andrews et al., 2021). The tract runs within the perithalamic white matter to the level of the lateral geniculate nucleus. The radiation extends from the lateral geniculate nucleus to the occipital cortex.

*Olfactory tracts:* The olfactory pathway in the dog was dissected as previously described (Andrews et al., 2022). These include the olfactory occipital tract (OOT) which extends between the dorsal aspect of the olfactory bulb and the occipital pole, the olfactory piriform tract (OPT) which extends from the olfactory bulb through the anterior internal capsule to the piriform cortex, olfactory limbic tract (OLT) which extends through the limbic system to terminate in the frontal lobe, the olfactory corticospinal tract (OCST) which extends from the ventral aspect of the olfactory bulb, through the anterior internal capsule, mesencephalon, and medulla oblongata and into the spinal cord, and the olfactory entorhinal tract (OET) which extends to the entorhinal cortex directly from the bulb and peduncle.

#### 3.2.2 Association tracts

*Cingulum:* The cingulum lies within the cingulate gyrus with fibers extending to frontal, sensory-motor, parietal, temporal, and occipital regions. It was dissected by placing multiple seed regions within the cingulate white matter to ensure both long and short fiber components were incorporated.

*Inferior Fronto-occipital Fasciculus:* The IFOF connects the ventromedial occipital cortex to the frontal lobe. It runs between these regions within the internal capsule medial to the SLF and lateral to the optic radiation. It was dissected by placing a seed region within the occipital lobe and only including fibers running to the frontal lobe via the internal capsule.

*Superior Fronto-occipital Fasciculus:* The SFOF extends between the ventral occipital cortex and frontal lobe via a more medial course than the IFOF. It extends dorsal to the thalamus and then lateral to the caudate nucleus. It has been described in the canine by Muratoff (1893) who termed it the subcallosal fascicle (Muratoff, 1893; Pascalau et al., 2016) It was dissected by placing seed regions in the occipital and frontal lobes and including pathways extending along dorsal aspect of the thalamus.

*Inferior Longitudinal Fasciculus:* This caudoventrally situated association pathway connects the ipsilateral occipital and temporal lobes, and is thought to participate in visual recognition. The tract was identified using seed regions placed in the occipital and ventral temporal regions.

*Superior Longitudinal Fasciculus:* This tract represents a series of long-range and short-range (arcuate) fibers that connect ipsilateral gyri within the cerebral hemispheres. The tract is situated dorsally and lateral to the centrum semiovale. Here, this tract was identified using a single arcuate ROI placed within the dorsal aspect of temporal lobe on the dorsal plane.

*Uncinate Fasciculus:* The UF connects the frontal and temporal lobes. It was dissected by identifying fibers that ran between seed regions within the frontal and ventral temporal white matter. The tract has a lateralized location lying within the proreus gyral white matter. It has a similar course to that identified on white matter dissection (Pascalau et al., 2016).

#### 3.2.3 Commissural tracts

Corpus callosum: The corpus collosum is the largest commissural tract connecting cortical areas in the right and left hemispheres. Dissection was performed as previously described (Johnson, Barry, et al., 2019). A single seed region placed on the corpus callosum on the mid-sagittal image was used to identify this pathway.

#### 3.2.4 Cerebellar tracts

*Caudal cerebellar peduncle:* The caudal cerebellar peduncle contains white matter pathways that extend between the cerebellum and dorsal spinal cord. It was dissected by placing seed regions over the caudal cerebellar peduncle and including only tracts that extended to the spinal cord.

*Middle cerebellar peduncle:* The middle cerebellar peduncle contains white matter pathways that extend from the cerebellum to the pons and mesencephalon. This pontocerebellar tract was dissected using a seed region placed over the middle cerebellar peduncle.

*Rostral cerebellar peduncle:* The rostral cerebellar peduncle contains white matter tracts that extend both to cerebral cortex via the thalamus and to the ventral spinal cord. These were dissected by placing seed regions over the rostral cerebellar peduncle and including tracts that extended to the level of the cerebral cortex.

### 3.3 Generation of atlas schematics

**Fig. 2.**
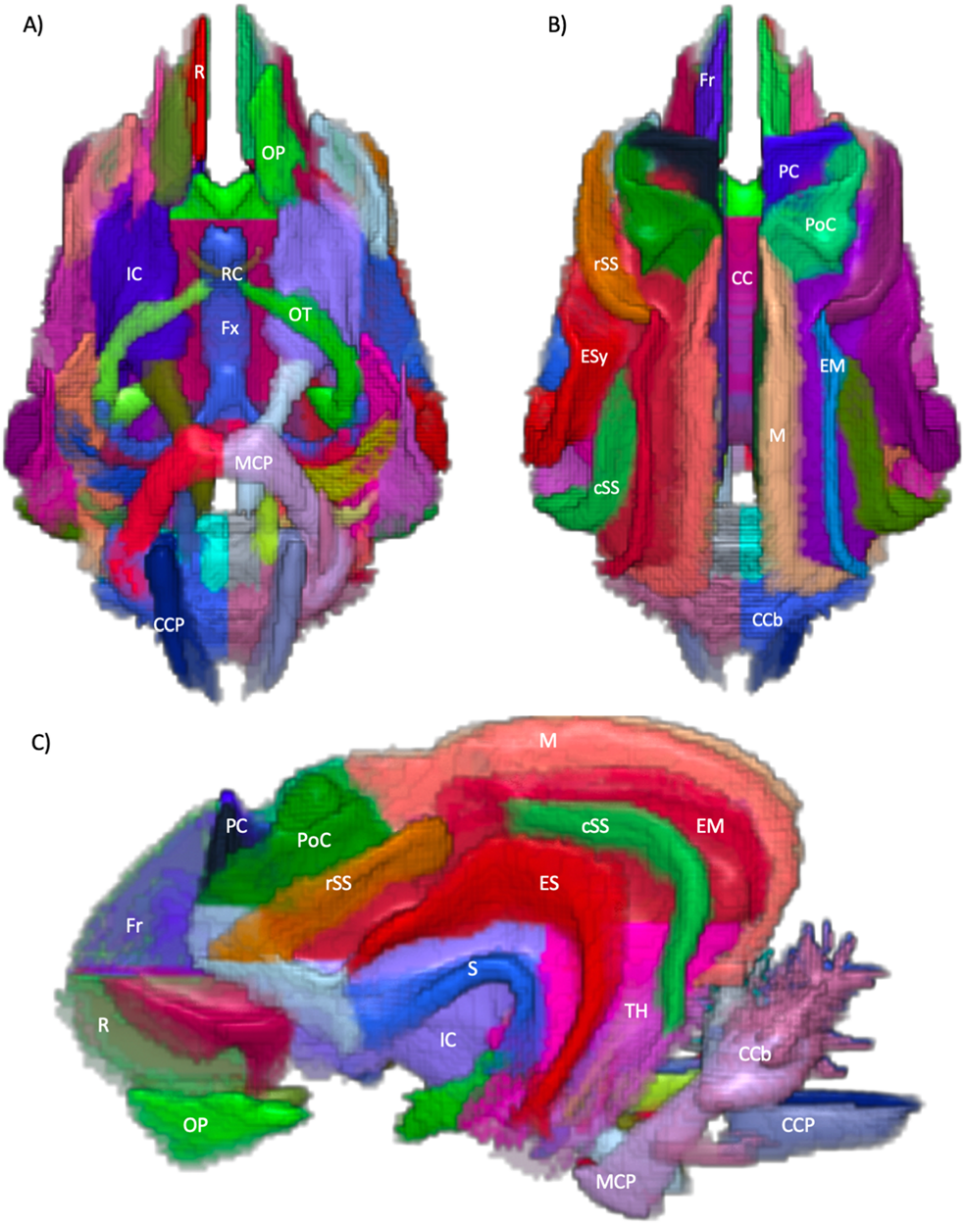
3-dimentional render images of the regional white matter atlas. (A) ventral view, (B) dorsal view, (C) left lateral view. CC = Corpus Callosum, CCb = Caudal cerebellum, CCP = Caudal cerebellar peduncle, ES = Ectosylvian, EM = Ectomarginal, Fr = Frontal, Fx = Fornix, IC = Internal capsule, M = Marginal, MCP = Middle cerebellar peduncle, OP = Olfactory peduncle, OT = Optic tract, PC = pre-cruciate, PoC = post-cruciate, R = Rectus, RC = Rostral commissure, S = Sylvian, cSS = Caudal Suprasylvian, rSS = Rostral Suprasylvian and TH = Temporal horn. Figure generated using MRIcroGL (64-bit OSX Cocoa).

**Fig. 3.**
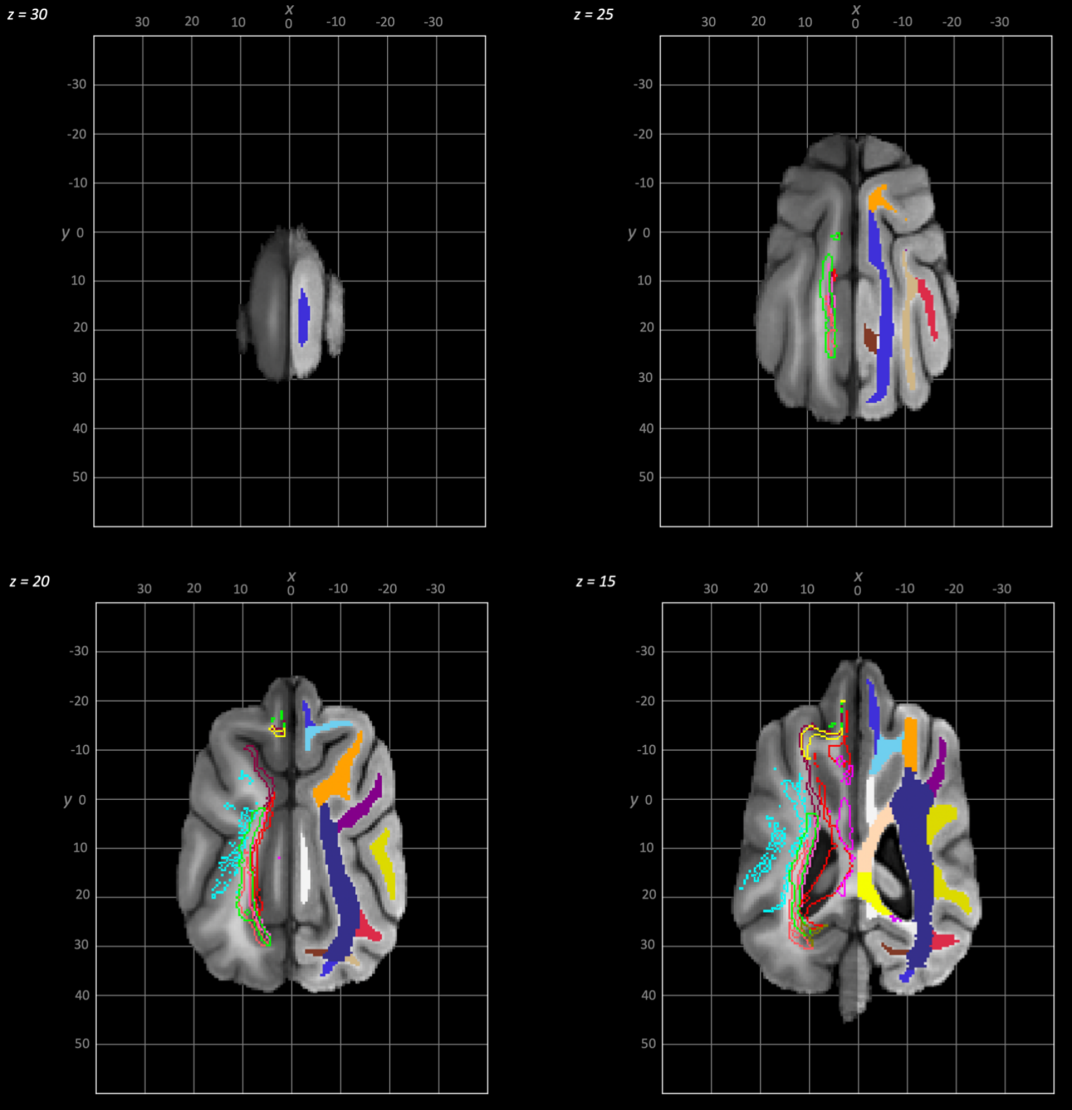
Dorsal to ventral dorsal plane slices of the population average brain template from z-30 to z-15. In each section, the left hemisphere demonstrates the white matter regions on the T1-weighted population average template and the right hemisphere demonstrates the white matter tracts dissected from the population average tractogram overlain on the population average FA map. The key for all regions is provided in Figure 8.

**Fig. 4.**
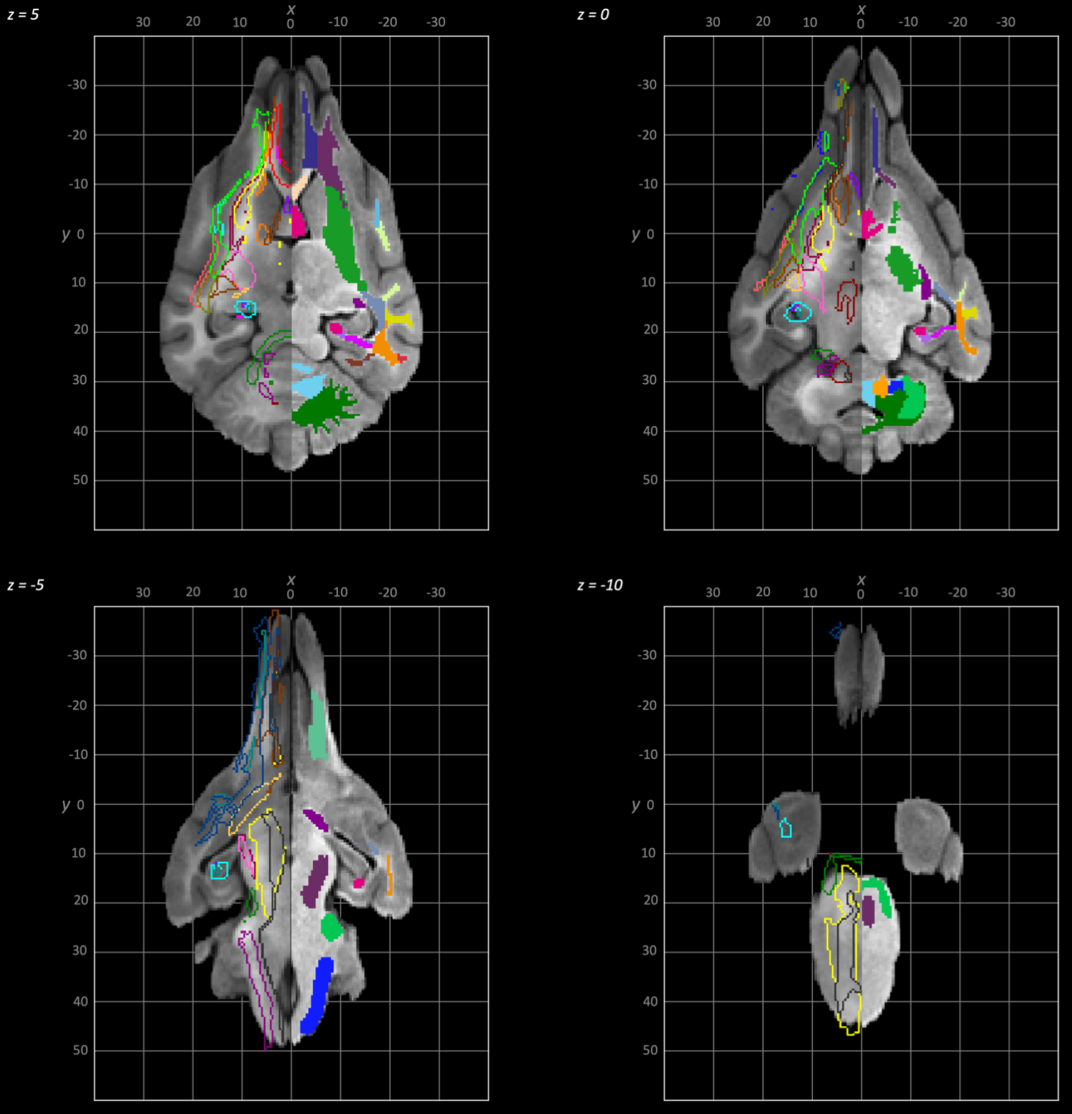
Dorsal to ventral dorsal plane slices of the population average brain template from z-5 to z-10. In each section, the left hemisphere demonstrates the white matter regions on the T1-weighted population average template and the right hemisphere demonstrates the white matter tracts dissected from the population average tractogram overlain on the population average FA map. The key for all regions is provided in Figure 8.

**Fig. 5.**
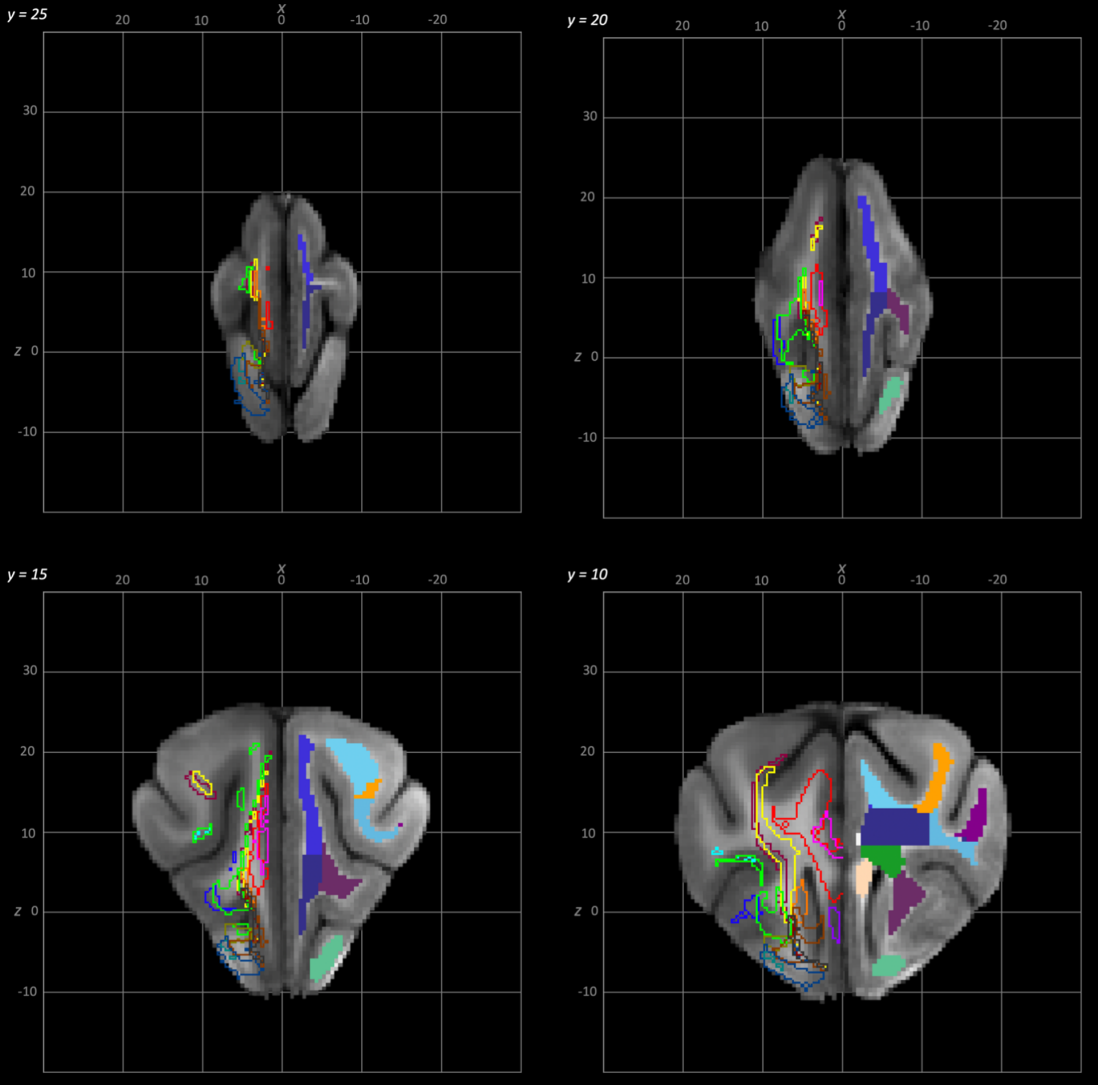
Rostral to caudal transverse slices of the population average brain template from y-25 to y-10. In each section, the left hemisphere demonstrates the white matter regions on the T1-weighted population average template and the right hemisphere demonstrates the white matter tracts dissected from the population average tractogram. The atlas key is provided in Figure 8.

**Fig. 6.**
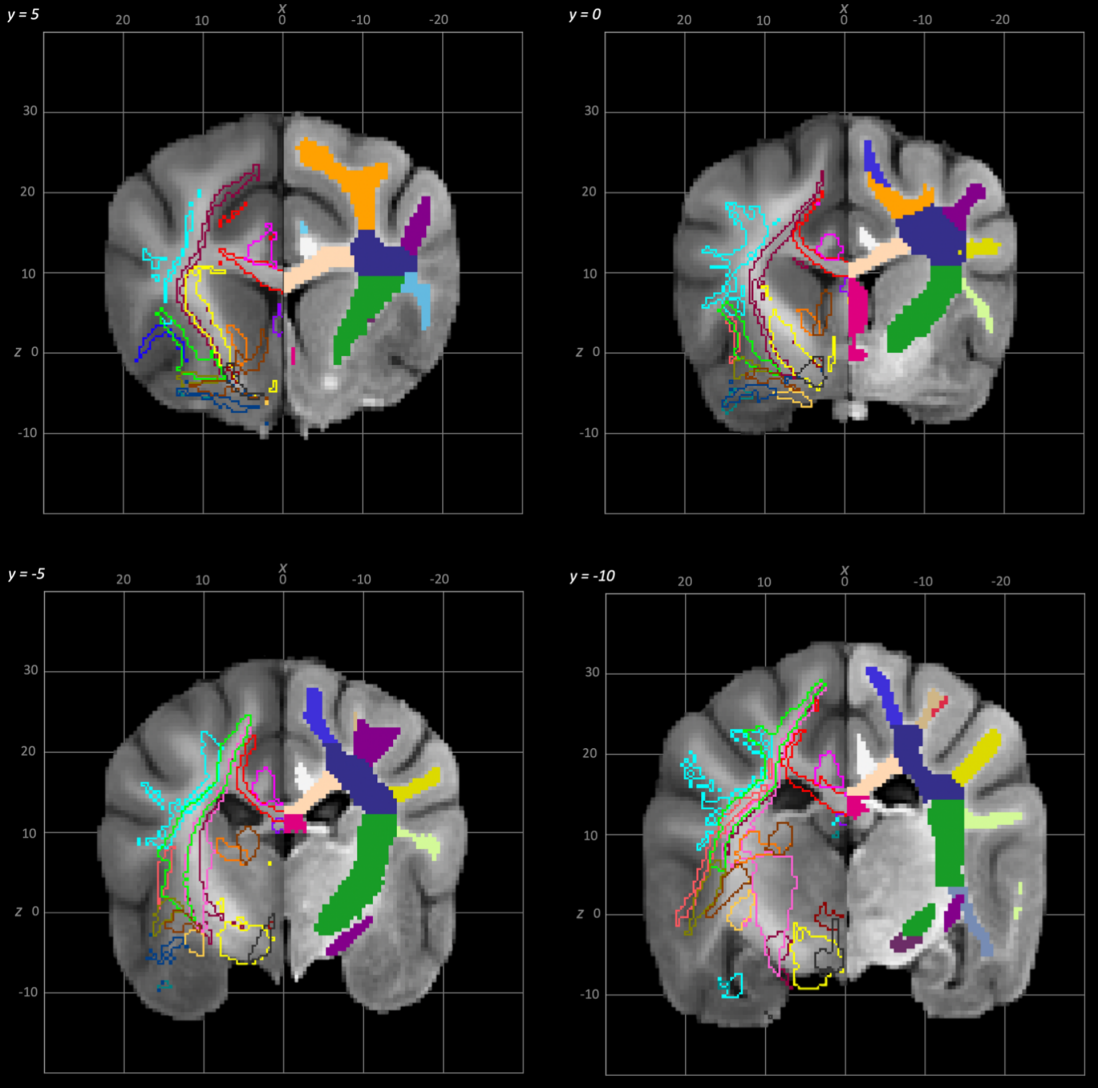
Rostral to caudal transverse slices of the population average brain template from y-5 to y-10. In each section, the left hemisphere demonstrates the white matter regions on the T1-weighted population average template and the right hemisphere demonstrates the white matter tracts dissected from the population average tractogram. The atlas key is provided in Figure 8.

**Fig. 7.**
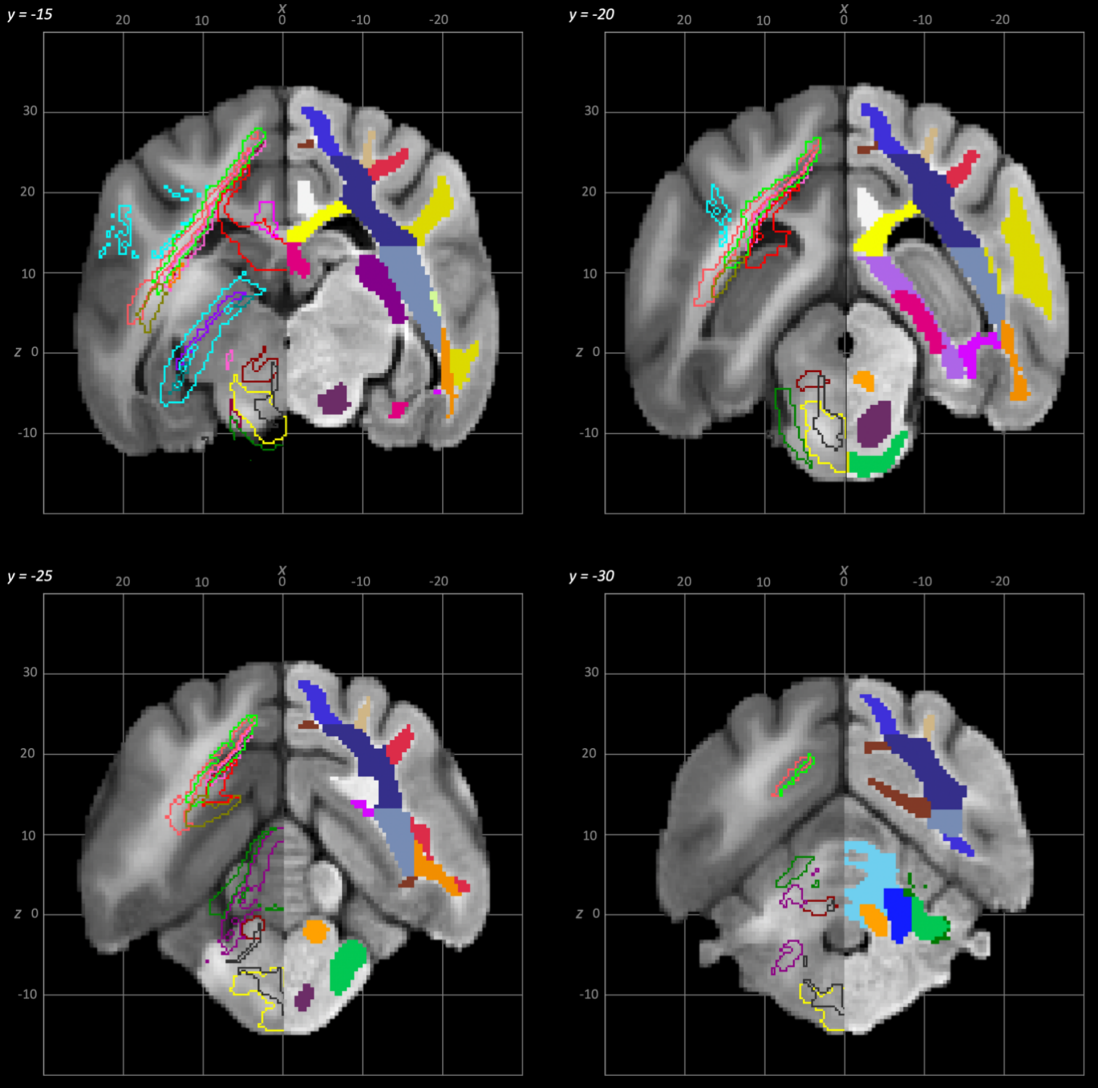
Rostral to caudal transverse slices of the population average brain template from y-15 to y-30. In each section, the left hemisphere demonstrates the white matter regions on the T1-weighted population average template and the right hemisphere demonstrates the white matter tracts dissected from the population average tractogram. The atlas key is provided in Figure 8.

**Fig. 8.**
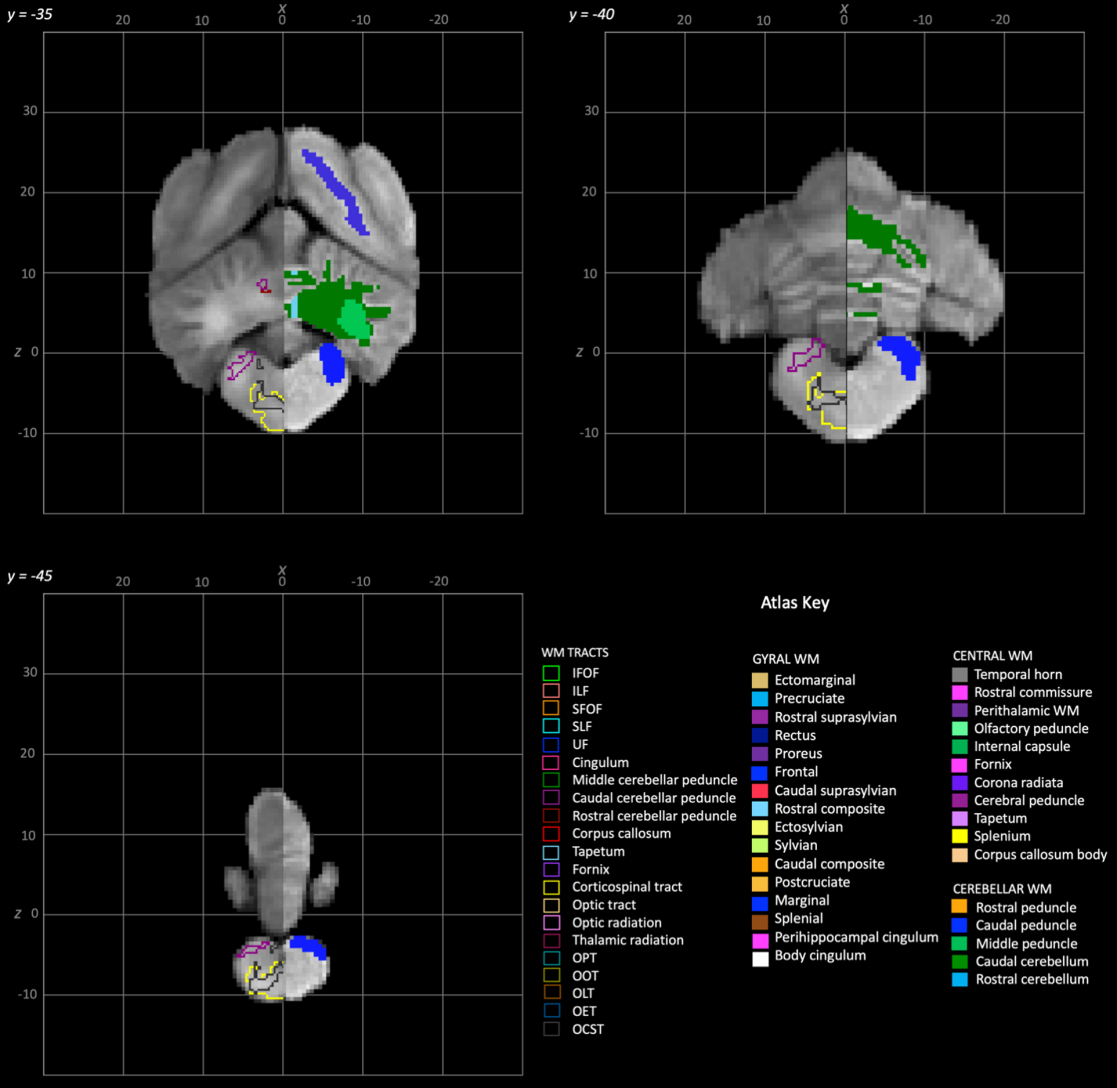
Rostral to caudal transverse slices of the population average brain template from y-35 to y-45. In each section, the left hemisphere demonstrates the white matter regions on the T1-weighted population average template and the right hemisphere demonstrates the white matter tracts dissected from the population average tractogram. The atlas key is provided in the bottom right of the figure and relates to Figures 3–8.

The following schematics of the atlas were generated; 3D images are provided in Figure 2 (figure generated using MRIcroGL (64-bit OSX Cocoa)), dorsal plane images are provided in Figures 3 and 4 and transverse plane images are provided in Figures 5 to 8 (figures generated with FSLeyes and Powerpoint).

## 4 Discussion

In this study, we have created a canine white matter atlas based on a population average template of 30 normal mesaticephalic dogs. This atlas was created via manual segmentation of white matter in accordance with previously published resources (Czeibert, Andics, et al., 2019; Fletcher & Saveraid, 2009; Pascalau et al., 2016) in order to create mask files that can be imported for use with other MRI studies. In addition, using deterministic tractography we have characterized the location of major functional white matter tracts within the brain in living subjects. These files have been made available for download so that they can be used by investigators for future canine neuroimaging research. This white matter atlas is aligned with the previously published canine cortical atlas, in which cortical regions were mapped according to myeloarchitectonic parcellations (Johnson, Luh, et al., 2020). Given the increasing reliance on canines in comparative studies of brain structure and function (Bunford et al., 2017; Dewey et al., 2020; Horschler & MacLean, 2019) and the use of MRI for clinical diagnostic purposes, it is the hope of the authors that this atlas will provide a standard reference for canine white matter tracts.

Several atlases based on MRI imaging now exist for the canine brain in which T1-weighted images provide good resolution of and segmentation between grey and white matter in much of the brain (Czeibert, Baksa, et al., 2019; Datta et al., 2012; Fletcher & Saveraid, 2009). However, since axons from different pathways converge at various points within the brain, these atlases do not always distinguish separate axonal pathways. This is particularly the case in the brainstem, which conveys numerous distinct pathways between the brain and spinal cord important in motor initiation and feedback, and in which grey and white matter are indistinct. To delineate specific white matter pathways, DTI has been used in conjunction with tractography techniques which help identify several distinct pathways (Anaya García et al., 2015; Berns et al., 2015; Cook et al., 2018; Jacqmot et al., 2013, 2017). These studies have provided valuable information in establishing the use of DTI in identifying functional white matter tracts. Our atlas extends these previous findings to examine functional white matter tracts in living subjects, and employs a larger population, thus limiting artifacts due to small sample size or post-mortem changes. To date, several white matter atlases have been created using DTI for humans (Catani & Thiebaut de Schotten, 2008; Mori et al., 2011; Oishi et al., 2008; Y. Zhang et al., 2010) and non-human primates (Calabrese et al., 2015; Oishi et al., 2011; Zakszewski et al., 2014), and similar atlases now exist for other species including feline (Johnson, Pascalau, et al., 2020), rodent (Harsan et al., 2010; Jiang & Johnson, 2011), equine (Boucher et al., 2020), and ovine (Nitzsche et al., 2015; Pieri et al., 2019) brains. The generation of this canine brain atlas complements these studies and provides a comprehensive reference for comparative studies between canines and other species.

Comparative studies of white matter tracts demonstrate significant differences in the organization of brains across species. Hecht et al. (2015) showed that connectivity within frontal areas of the brain differs between humans and primates, such that in primates there was a larger connectivity with regions involved in motor planning, whereas pathways associated with executive functions were more developed in humans. Similarly, comparisons of white matter tracts in dogs and cats have revealed that the limbic system occupies a greater portion of the brain in cats, whereas frontoparietal connections present in dogs were not found in cats (Jacqmot et al., 2017). These results have important implications for understanding structure-function relationships and for elucidating pathologies such as affective disorders that manifest differently across species. Consequently, these observations are important in considering animal models of disease in research.

Numerous studies in humans have now demonstrated the utility of DTI tractography in mapping white matter pathways in healthy and diseased brains. For example, DTI can be applied at various stages across the lifespan, from fetal to late adult life stages, to examine temporal and regional changes in functional white matter pathways (Lebel et al., 2012; Oishi et al., 2019). In humans, these studies demonstrate that white matter expands rapidly up to approximately 3 years of age, with continued growth of many tracts into early adulthood. In contrast, grey matter volume decreases after the age of 5 (Lebel & Deoni, 2018). These studies also demonstrate that individual white matter tracts differ in their rate of development, such that more caudal regions of white matter reach maximal fractional anisotropy before those lying rostrally (Lebel et al., 2012). In children, fractional anisotropy has been shown to correlate with reading ability (Deutsch et al., 2005). With age, fractional anisotropy decreases more rapidly in white matter tracts in frontal areas of the human brain involved in executive planning and reasoning, consistent with decline in executive functions that accompanies normal aging (Gunning-Dixon et al., 2009; Lebel et al., 2010; O’Sullivan et al., 2001; Ota & Shah, 2022). Patients with Alzheimer’s disease, a disease diagnosed on the basis of early or severe cognitive decline, display consistent decline in volume and FA of white matter supplying frontotemporal and limbic regions (Chen et al., 2023; Talwar et al., 2021), and some evidence suggests that decline in FA values precedes anatomic loss of white matter, giving weight to DTI as a promising tool to identify individuals most at risk for decline (Hugenschmidt et al., 2008). Canines display similar cognitive decline with age, and similar histopathological changes within the brain (Head, 2013). Recently, DTI-based studies of white matter in aged canines revealed, as in humans, that these animals have reduced fractional anisotropy in frontotemporal brain regions associated with executive functions (Barry et al., 2021). Analogous to humans, canines can suffer from dementia as they age (Dewey et al., 2019; Head, 2013), positioning dogs as promising clinical models for Alzheimer’s disease.

Canines suffer from several other neurological diseases in line with human counterparts, including epilepsy (Charalambous et al., 2023; Löscher, 2022), degenerative diseases (Coates & Wininger, 2010; Story et al., 2020), dyskinesias (Urkasemsin & Olby, 2014), neoplasia (Hicks et al., 2017; A. D. Miller et al., 2019), and inflammatory diseases (Cornelis et al., 2019), and these similarities cement their place as naturally-occurring pre-clinical models for human disease. For many neurological diseases, DTI has provided insights into changes that occur in white matter pathways, permitting structure-function relationships to be established. DTI studies are also useful in drawing comparisons between human and canine neurological disease. Developmental abnormalities of the corpus callosum, for example, which constitute common malformations of the human brain, are associated with neurodevelopmental delay (Probst, 1901; Rakic & Yakovlev, 1968; Sztriha, 2005). In these patients, DTI studies indicate reduced densities of interhemispheric fibers, and instead, aberrant, long-range pathways, named “Probst bundles,” that remain in one hemisphere (Bénézit et al., 2015; Yeon Kim et al., 2004). Corpus callosal abnormalities are also detected in canines (Gonçalves et al., 2014), and DTI imaging of canine corpus callosum agenesis has indicated similarly few interhemispheric fibers, with large numbers of Probst bundles (Johnson, Barry, et al., 2019; Wang-Leandro et al., 2018).

Canine degenerative myelopathy, a disease considered analogous to amyotrophic lateral sclerosis (ALS) in humans, is characterized by loss of motor neurons, resulting in progressive general proprioceptive ataxia and paresis, progressing to plegia (Coates & Wininger, 2010; Nardone et al., 2016). In both the canine disease and a familial form of the human disease, genetic mutations of the superoxide dismutase I (*SOD1*) gene have been isolated (Awano et al., 2009; Boillée et al., 2006). These mutations have been associated with a mutant, gain-of-function enzyme that leads to protein mis-folding, aggregate deposition, and loss of neuronal integrity in human patients (Boillée et al., 2006). In both species, DTI has demonstrated reduced fractional anisotropy within the spinal cord (Johnson et al., 2021; Nair et al., 2010) findings consistent with loss of axonal load. These studies highlight the use of fractional anisotropy and DTI in assessing in vivo changes in white matter function that may facilitate disease staging and prognosis, and emphasize the utility of canines in modeling human disease.

DTI is also used in humans for surgical planning to remove brain tumors (Costabile et al., 2019; Henderson et al., 2020) and epileptic foci (Sivakanthan et al., 2016; Yogarajah & Duncan, 2008) to avoid neuronal connections critical for cognitive functions such as language and reasoning, underscoring the clinical potential for DTI as a non-invasive method for assessing white matter function in situ. While this approach has not been used for canines, the disease processes are similar. The rate of epilepsy in humans and dogs is similar, and these species are prone to refractory disease at similar rates (Charalambous et al., 2023; Löscher, 2022). Studies of glioma in canines have found genetic similarities with human patients (Amin et al., 2020). Therefore, canines are an ideal species for comparative studies of white matter function and pathology in the human counterpart.

DTI has also been used to investigate previously unidentified pathways. Using a seed function to delineate white matter passing through specific regions of brain, long-range functional pathways may be identified that are not easily discerned in studies of white matter dissection in cadavers. Recently, DTI has been employed in canines to identify an extensive complex of pathways between the olfactory bulb and other brain regions, including hitherto undescribed connections to the occipital lobe (Andrews et al., 2022). Given increased interest employing canines to detect various health disorders such as SARS-CoV2 (Jendrny et al., 2021), these results provide a foundation for screening dogs to identify those animals most suitable to perform olfactory-based tasks.

While our atlas was generated with reference to several other published sources incorporating gross dissection techniques, histology and imaging, borders between adjacent white matter regions are poorly defined. To facilitate comparative studies, we have attempted, where possible, to use similar borders and nomenclature to those used in other atlases (Mori et al., 2011; Zakszewski et al., 2014). However arbitrary borders between structures do not necessarily represent functionally discrete regions: for example, the corona radiata, internal capsule, and cerebral peduncles represent a continuous white matter pathway in which several tracts are combined. To help delineate these various pathways, we employed deterministic tractography to identify established white matter tracts. In doing so, this atlas does not identify novel tracts, and therefore is not an exhaustive list of functional connectivity within the brain.

Another limitation of this atlas is that the spatial resolution is limited by a voxel size of 1.5 mm^3^. Since deterministic tractography depends on creating tensors from eigenvectors within a single voxel, regions containing compact, small, non-myelinated fibers with various orientations may not be differentiated accurately due to volume averaging artifact. While the use of a sizeable population may minimize such error, studies of gyral white matter in humans indicate low fractional anisotropy within the corona radiata and centrum semiovale (Brander et al., 2010; Hakulinen et al., 2012). Similarly, in our atlas, fractional anisotropy within the gyral white matter frequently corresponded to only a portion of the white matter identified in the population average T1w template. Accordingly, variability in the size and direction of local axonal connections within individual gyri, in which small, unmyelinated fibers are numerous, may create incongruities in calculated fractional anisotropy. For this atlas we elected to use fractional anisotropy to identify individual gyral white matter initially, and to use the T1w template to complete the manual segmentations for each gyral mask. A significant limitation of deterministic tractography is the inability to resolve pathways where there is significant crossing of axon bundles, or to distinguish between axons that cross and those that converge but stay separate (so-called “kissing” artifact) (Dell’Acqua & Tournier, 2019). As a result, while the individual masks are anatomically correct for each gyrus, functional pathways within in gyral white matter remain difficult to quantify, and false negatives are possible. Therefore, an important consideration within these studies is that absence of a discernable white matter tract in DTI tractography does not necessarily imply that none exists. In addition, since the detection of white matter pathways depends on the degree of diffusivity in each voxel, smaller fiber tracts within the vicinity may be masked by larger fiber tracts with greater fractional anisotropy, and therefore remain undetected. Further, in regions where fractional anisotropy is low, models of best fit may result in false positives. Notwithstanding these limitations, tractography has advantages over previous histologic methods that required invasive interventions and processing post-mortem specimens, where fibers of passage may be incorrectly identified as a source of innervation for a particular region of interest (Gorbachevskaya, 2014; Sakai et al., 1993). Future work could consider utilizing post-mortem brain imaging where prolonged acquisition times allow for ultra-high resolution DTI data acquisition (Berns et al., 2015; Cook et al., 2018; K. L. Miller et al., 2012).

In summary, we have developed a detailed white matter atlas of the canine brain based on a T1w population average template, using DTI to manually delineate white matter tracts. In addition, individual projection pathways were identified using tractography. This atlas provides a basis for future studies in comparative anatomy and neurological disease. This atlas allows for the addition of novel tracts and refined masks as new findings arise. It is anticipated that these tract files and manual segmentations will facilitate interpretation of quantitative studies of white matter tracts in clinical and research settings.

## Data and Code Availability

The final atlas is available, open source at: https://doi.org.10.7298/2w4q-8j27 and https://hdl.handle.net/1813/113846

## Author Contributions

Fiona M. Inglis: Study design, manual delineation of white matter regions, and manuscript preparation. Paul A. Taylor: Creation and editing of atlas files and review of manuscript. Erica F. Andrews: Data processing and review of manuscript. Raluca Pascalau: Review of white matter anatomy and expert input into resulting white matter segmentation and review of manuscript. Henning U. Voss: MR imaging, diffusion tensor imaging processing, and review of manuscript. Daniel R. Glen: Data analysis and review of manuscript. Philippa J. Johnson: Subject recruitment, MR imaging, study design, deterministic tractography dissections, figure creation, review of white matter segmentation, and review of manuscript.

## Declaration of Competing Interest

Paul A. Taylor and Daniel R. Glen were supported by the NIMH Intramural Research Programs (ZICMH002888) of the NIH (HHS, USA).

## Supplementary Materials

Supplementary material for this article is available with the online version here: https://doi.org/10.1162/imag_a_00276

## Supplementary Material

Supplementary Material

## References

[b138] Adrianov, O. S. (2010). Atlas of the canine brain. S.l.: NPP BOOKS.

[b1] Alexander, A. L., Lee, J. E., Lazar, M., Boudos, R., DuBray, M. B., Oakes, T. R., Miller, J. N., Lu, J., Jeong, E. K., McMahon, W. M., Bigler, E. D., & Lainhart, J. E. (2007). Diffusion tensor imaging of the corpus callosum in Autism. NeuroImage, 34(1), 61–73. 10.1016/j.neuroimage.2006.08.032 17023185

[b2] Amin, S. B., Anderson, K. J., Boudreau, C. E., Martinez-Ledesma, E., Kocakavuk, E., Johnson, K. C., Barthel, F. P., Varn, F. S., Kassab, C., Ling, X., Kim, H., Barter, M., Lau, C. C., Ngan, C. Y., Chapman, M., Koehler, J. W., Long, J. P., Miller, A. D., Miller, C. R., … Verhaak, R. G. W. (2020). Comparative molecular life history of spontaneous canine and human gliomas. Cancer Cell, 37(2), 243.e7–257.e7. 10.1016/j.ccell.2020.01.004 32049048 PMC7132629

[b3] Anaya García, M. S., Hernández Anaya, J. S., Marrufo Meléndez, O., Velázquez Ramírez, J. L., & Palacios Aguiar, R. (2015). In vivo study of cerebral white matter in the dog using diffusion tensor tractography. Veterinary Radiology & Ultrasound, 56(2), 188–195. 10.1111/VRU.12211 25288360 PMC4409102

[b4] Andersson, J. L. R., Skare, S., & Ashburner, J. (2003). How to correct susceptibility distortions in spin-echo echo-planar images: Application to diffusion tensor imaging. NeuroImage, 20(2), 870–888. 10.1016/S1053-8119(03)00336-7 14568458

[b5] Andersson, J. L. R., & Sotiropoulos, S. N. (2016). An integrated approach to correction for off-resonance effects and subject movement in diffusion MR imaging. NeuroImage, 125, 1063–1078. 10.1016/j.neuroimage.2015.10.019 26481672 PMC4692656

[b6] Andics, A., & Miklósi, Á. (2018). Neural processes of vocal social perception: Dog-human comparative fMRI studies. Neuroscience and Biobehavioral Reviews, 85, 54–64. 10.1016/j.neubiorev.2017.11.017 29287629

[b7] Andrews, E. F., Jacqmot, O., Gomes Espinheira, M. F. N. C., Sha, M. F., Niogi, S. N., & Johnson, P. J. (2021). Characterizing the canine and feline optic pathways in vivo with diffusion MRI. Veterinary Ophthalmology, 25(Suppl. 1), 60–71. 10.1111/vop.12940 34784441

[b8] Andrews, E. F., Pascalau, R., Horowitz, A., Lawrence, G. M., & Johnson, P. J. (2022). Extensive connections of the canine olfactory pathway revealed by tractography and dissection. Journal of Neuroscience, 42(33), 6392–6407. 10.1523/JNEUROSCI.2355-21.2022 35817576 PMC9398547

[b9] Awano, T., Johnson, G. S., Wade, C. M., Katz, M. L., Johnson, G. C., Taylor, J. F., Perloski, M., Biagi, T., Baranowska, I., Long, S., March, P. A., Olby, N. J., Shelton, G. D., Khan, S., O’Brien, D. P., Lindblad-Toh, K., & Coates, J. R. (2009). Genome-wide association analysis reveals a *SOD1* mutation in canine degenerative myelopathy that resembles amyotrophic lateral sclerosis. Proceedings of the National Academy of Sciences of the United States of America, 106(8), 2794–2799. 10.1073/pnas.0812297106 19188595 PMC2634802

[b10] Barry, E. F., Loftus, J. P., Luh, W. M., Leon de, J. M., Niogi, S. N., & Johnson, P. J. (2021). Diffusion tensor-based analysis of white matter in the healthy aging canine brain. Neurobiology of Aging, 105, 129–136. 10.1016/j.neurobiolaging.2021.04.021 34062488

[b11] Basser, P. J., & Jones, D. K. (2002). Diffusion-tensor MRI: Theory, experimental design and data analysis—A technical review. NMR in Biomedicine, 15(7–8), 456–467. 10.1002/nbm.783 12489095

[b12] Bazin, P. L., Ye, C., Bogovic, J. A., Shiee, N., Reich, D. S., Prince, J. L., & Pham, D. L. (2011). Direct segmentation of the major white matter tracts in diffusion tensor images. NeuroImage, 58(2), 458–468. 10.1016/j.neuroimage.2011.06.020 21718790 PMC3159825

[b13] Beaulieu, C. (2002). The basis of anisotropic water diffusion in the nervous system—A technical review. NMR in Biomedicine, 15(7–8), 435–455. 10.1002/nbm.782 12489094

[b14] Behrens, T. E. J., Berg, H. J., Jbabdi, S., Rushworth, M. F. S., & Woolrich, M. W. (2007). Probabilistic diffusion tractography with multiple fibre orientations: What can we gain? NeuroImage, 34(1), 144–155. 10.1016/j.neuroimage.2006.09.018 17070705 PMC7116582

[b15] Behrens, T. E. J., Woolrich, M. W., Jenkinson, M., Johansen-Berg, H., Nunes, R. G., Clare, S., Matthews, P. M., Brady, J. M., & Smith, S. M. (2003). Characterization and propagation of uncertainty in diffusion-weighted MR imaging. Magnetic Resonance in Medicine, 50(5), 1077–1088. 10.1002/mrm.10609 14587019

[b16] Bénézit, A., Hertz-Pannier, L., Dehaene-Lambertz, G., Monzalvo, K., Germanaud, D., Duclap, D., Guevara, P., Mangin, J.-F., Poupon, C., Moutard, M.-L., & Dubois, J. (2015). Organising white matter in a brain without corpus callosum fibres. Cortex, 63, 155–171. 10.1016/j.cortex.2014.08.022 25282054

[b17] Berns, G. S., Cook, P. F., Foxley, S., Jbabdi, S., Miller, K. L., & Marino, L. (2015). Diffusion tensor imaging of dolphin brains reveals direct auditory pathway to temporal lobe. Proceedings of the Royal Society B: Biological Sciences, 282(1811), 20151203. 10.1098/rspb.2015.1203 PMC4528565 26156774

[b18] Blackwell, E. J., Bradshaw, J. W. S., & Casey, R. A. (2013). Fear responses to noises in domestic dogs: Prevalence, risk factors and co-occurrence with other fear related behaviour. Applied Animal Behaviour Science, 145(1–2), 15–25. 10.1016/j.applanim.2012.12.004

[b19] Boch, M., Wagner, I. C., Karl, S., Huber, L., & Lamm, C. (2023). Functionally analogous body- and animacy-responsive areas are present in the dog (*Canis familiaris*) and human occipito-temporal lobe. Communications Biology, 6(1), 645. 10.1038/s42003-023-05014-7 37369804 PMC10300132

[b20] Boillée, S., Velde Vande, C., & Cleveland, D. W. W. (2006). ALS: A disease of motor neurons and their nonneuronal neighbors. Neuron, 52(1), 39–59. 10.1016/j.neuron.2006.09.018 17015226

[b21] Bonilha, L., Helpern, J. A., Sainju, R., Nesland, T., Edwards, J. C., Glazier, S. S., & Tabesh, A. (2013). Presurgical connectome and postsurgical seizure control in temporal lobe epilepsy. Neurology, 81(19):1704–1710. 10.1212/01.wnl.0000435306.95271.5f 24107863 PMC3812102

[b22] Boucher, S., Arribarat, G., Cartiaux, B., Lallemand, E. A., Péran, P., Deviers, A., & Mogicato, G. (2020). Diffusion tensor imaging tractography of white matter tracts in the equine brain. Frontiers in Veterinary Science, 7, 1–10. 10.3389/fvets.2020.00382 32850994 PMC7406683

[b23] Brander, A., Kataja, A., Saastamoinen, A., Ryymin, P., Huhtala, H., Öhman, J., Soimakallio, S., & Dastidar, P. (2010). Diffusion tensor imaging of the brain in a healthy adult population: Normative values and measurement reproducibility at 3 T and 1.5 T. Acta Radiologica, 51(7), 800–807. 10.3109/02841851.2010.495351 20707664

[b24] Bunford, N., Andics, A., Kis, A., Miklósi, Á., & Gácsi, M. (2017). *Canis familiaris* as a model for non-invasive comparative neuroscience. Trends in Neurosciences, 40(7), 438–452. 10.1016/j.tins.2017.05.003 28571614

[b25] Bunford, N., Hernández-Pérez, R., Farkas, E. B., Cuaya, L. V., Szabó, D., Szabó, Á. G., Gácsi, M., Miklósi, Á., & Andics, A. (2020). Comparative brain imaging reveals analogous and divergent patterns of species and face sensitivity in humans and dogs. Journal of Neuroscience, 40(43), 8396–8408. 10.1523/JNEUROSCI.2800-19.2020 33020215 PMC7577605

[b26] Calabrese, E., Badea, A., Coe, C. L., Lubach, G. R., Shi, Y., Styner, M. A., & Johnson, G. A. (2015). A diffusion tensor MRI atlas of the postmortem rhesus macaque brain. NeuroImage, 117, 408–416. 10.1016/j.neuroimage.2015.05.072 26037056 PMC4512905

[b27] Catani, M., & Thiebaut de Schotten, M. (2008). A diffusion tensor imaging tractography atlas for virtual in vivo dissections. Cortex, 44(8), 1105–1132. 10.1016/j.cortex.2008.05.004 18619589

[b28] Charalambous, M., Fischer, A., Potschka, H., Walker, M. C., Raedt, R., Vonck, K., Boon, P., Lohi, H., Löscher, W., Worrell, G., Leeb, T., McEvoy, A., Striano, P., Kluger, G., Galanopoulou, A. S., Volk, H. A., & Bhatti, S. F. M. (2023). Translational veterinary epilepsy: A win-win situation for human and veterinary neurology. Veterinary Journal, 293, 105956. 10.1016/j.tvjl.2023.105956 36791876

[b29] Chen, Y., Wang, Y., Song, Z., Fan, Y., Gao, T., & Tang, X. (2023). Abnormal white matter changes in Alzheimer’s disease based on diffusion tensor imaging: A systematic review. Ageing Research Reviews, 87, 101911. 10.1016/j.arr.2023.101911 36931328

[b30] Coates, J. R., & Wininger, F. A. (2010). Canine degenerative myelopathy. Veterinary Clinics of North America—Small Animal Practice, 40(5), 929–950. 10.1016/j.cvsm.2010.05.001 20732599

[b31] Cook, P. F., Berns, G. S., Colegrove, K., Johnson, S., & Gulland, F. (2018). Postmortem DTI reveals altered hippocampal connectivity in wild sea lions diagnosed with chronic toxicosis from algal exposure. Journal of Comparative Neurology, 526(2), 216–228. 10.1002/cne.24317 28875534

[b32] Cornelis, I., Van Ham, L., Gielen, I., Decker De, S., & Bhatti, S. F. M. (2019). Clinical presentation, diagnostic findings, prognostic factors, treatment and outcome in dogs with meningoencephalomyelitis of unknown origin: A review. The Veterinary Journal, 244, 37–44. 10.1016/j.tvjl.2018.12.007 30825893

[b33] Correard, S., Plassais, J., Lagoutte, L., Botherel, N., Thibaud, J. L., Hédan, B., Richard, L., Lia, A. S., Delague, V., Mège, C., Mathis, S., Guaguère, E., Paradis, M., Vallat, J. M., Quignon, P., & André, C. (2019). Canine neuropathies: Powerful spontaneous models for human hereditary sensory neuropathies. Human Genetics, 138(5), 455–466. 10.1007/s00439-019-02003-x 30955094

[b34] Costabile, J. D., Alaswad, E., D’Souza, S., Thompson, J. A., & Ormond, D. R. (2019). Current applications of diffusion tensor imaging and tractography in intracranial tumor resection. Frontiers in Oncology, 9, 426. 10.3389/fonc.2019.00426 31192130 PMC6549594

[b35] Cox, R. W. (1996). AFNI: Software for analysis and visualization of functional magnetic resonance neuroimages. Computers and Biomedical Research, 29(3), 162–173. 10.1006/cbmr.1996.0014 8812068

[b36] Cox, R. W., Ashburner, J., Breman, H., Fissell, K., Haselgrove, C., Holmes, C. J., Lancaster, J. L., Rex, D. E., Smith, S. M., Woodward, J. B., & Strother, S. (2004). A (sort of) new image data format standard: NiFTI-1. In 10th Annual Meeting of the Organization for Human Brain Mapping, 22. https://nifti.nimh.nih.gov/nifti-1/documentation/hbm_nifti_2004.pdf

[b37] Czeibert, K., Andics, A., Petneházy, Ö., & Kubinyi, E. (2019). A detailed canine brain label map for neuroimaging analysis. Biologia Futura, 70(2), 112–120. 10.1556/019.70.2019.14 34554420

[b38] Czeibert, K., Baksa, G., Grimm, A., Nagy, S. A., Kubinyi, E., & Petneházy, Ö. (2019). MRI, CT and high resolution macro-anatomical images with cryosectioning of a Beagle brain: Creating the base of a multimodal imaging atlas. PLoS One, 14(3), 1–25. 10.1371/journal.pone.0213458 PMC6405067 30845177

[b39] Datta, R., Lee, J., Duda, J., Avants, B. B., Vite, C. H., Tseng, B., Gee, J. C., Aguirre, G. D., & Aguirre, G. K. (2012). A digital atlas of the dog brain. PLoS One, 7(12), e52140. 10.1371/journal.pone.0052140 23284904 PMC3527386

[b40] de Lahunta, A., Glass, E., & Kent, M. (2021a). Cerebellum. In de Lahunta’s Veterinary Neuroanatomy and Clinical Neurology (pp. 374–413). Elsevier. 10.1016/B978-0-323-69611-1.00013-X

[b41] de Lahunta, A., Glass, E., & Kent, M. (2021b). Upper Motor Neuron. In de Lahunta’s Veterinary Neuroanatomy and Clinical Neurology (pp. 230–245). Elsevier. 10.1016/B978-0-323-69611-1.00008-6

[b139] de Lahunta, A., Glass, E., & Kent, M. (2021c). Visual System. In de Lahunta’s Veterinary Neuroanatomy and Clinical Neurology (pp. 414–456). Elsevier. 10.1016/B978-0-323-69611-1.00014-1

[b42] Dell’Acqua, F., & Tournier, J.-. (2019). Modelling white matter with spherical deconvolution: How and why? NMR in Biomedicine, 32(4), e3945. 10.1002/nbm.3945 30113753 PMC6585735

[b43] Deutsch, G., Dougherty, R., R Bammer, WT Siok, JD Gabrieli, & B Wandell. (2005). Children’s reading performance is correlated with white matter structure measured by diffusion tensor imaging. Cortex, 41(6), 354–363. 10.1016/s0010-9452(08)70272-7 15871600

[b44] Dewey, C. W., Davies, E. S., Xie, H., & Wakshlag, J. J. (2019). Canine cognitive dysfunction: Pathophysiology, diagnosis, and treatment. Veterinary Clinics of North America—Small Animal Practice, 49(3), 477–499. 10.1016/j.cvsm.2019.01.013 30846383

[b45] Dewey, C. W., Rishniw, M., Johnson, P. J., Platt, S., Robinson, K., Sackman, J., & O’Donnell, M. (2020). Canine cognitive dysfunction patients have reduced total hippocampal volume compared with aging control dogs: A comparative magnetic resonance imaging study. Open Veterinary Journal, 10(4), 438–442. 10.4314/OVJ.V10I4.11 33614439 PMC7830179

[b46] Dickinson, P. J. (2020). Coronavirus Infection of the Central Nervous System: Animal models in the time of COVID-19. Frontiers in Veterinary Science, 7, 584673. 10.3389/fvets.2020.584673 33195610 PMC7644464

[b47] Dickinson, P. J., & Bannasch, D. L. (2020). Current understanding of the genetics of intervertebral disc degeneration. Frontiers in Veterinary Science, 7, 431. 10.3389/fvets.2020.00431 32793650 PMC7393939

[b48] Dreschel, N. A. (2010). The effects of fear and anxiety on health and lifespan in pet dogs. Applied Animal Behaviour Science, 125(3–4), 157–162. 10.1016/j.applanim.2010.04.003

[b49] Ekenstedt, K. J., & Oberbauer, A. M. (2013). Inherited epilepsy in dogs. Topics in Companion Animal Medicine, 28(2), 51–58. 10.1053/j.tcam.2013.07.001 24070682

[b50] Figini, M., Zucca, I., Aquino, D., Pennacchio, P., Nava, S., Marzio Di, A., Preti, M. G., Baselli, G., Spreafico, R., & Frassoni, C. (2015). In vivo DTI tractography of the rat brain: An atlas of the main tracts in Paxinos space with histological comparison. Magnetic Resonance Imaging, 33(3), 296–303. 10.1016/j.mri.2014.11.001 25482578

[b51] Flegel, T., Kornberg, M., Mühlhause, F., Neumann, S., Fischer, A., Wielaender, F., König, F., Pakozdy, A., Quitt, P. R., Trapp, A. M., Jurina, K., Steffen, F., Rentmeister, K. W., Flieshardt, C., & Dietzel, J. (2021). A retrospective case series of clinical signs in 28 Beagles with Lafora disease. Journal of Veterinary Internal Medicine, 35(5), 2359–2365. 10.1111/jvim.16255 34486182 PMC8478043

[b52] Fletcher, T. F., & Saveraid, T. (2009). Canine brain MRI atlas. University of Minnesota College of Veterinary Medicine, February 2018, 1–2. http://vanat.cvm.umn.edu/mriBrainAtlas

[b53] Freund, P., Wheeler-Kingshott, C., Jackson, J., Miller, D., Thompson, A., & Ciccarelli, O. (2010). Recovery after spinal cord relapse in multiple sclerosis is predicted by radial diffusivity. Multiple Sclerosis, 16(10), 1193–1202. 10.1177/1352458510376180 20685759 PMC2951108

[b54] Garafalo, A. V., Cideciyan, A. V., Héon, E., Sheplock, R., Pearson, A., Yu WeiYang, C., Sumaroka, A., Aguirre, G. D., & Jacobson, S. G. (2020). Progress in treating inherited retinal diseases: Early subretinal gene therapy clinical trials and candidates for future initiatives. Progress in Retinal and Eye Research, 77, 100827. 10.1016/j.preteyeres.2019.100827 31899291 PMC8714059

[b55] Gonçalves, R., Volk, H., Smith, P. M., Penderis, J., Garosi, L., Mackillop, E., Stefani de, A., Cherubini, G., & Mcconnell, J. F. (2014). Corpus callosal abnormalities in dogs. Journal of Veterinary Internal Medicine, 28(4), 1275–1279. 10.1111/jvim.12371 24839863 PMC4857934

[b56] Gorbachevskaya, A. I. (2014). Organization of projections of the rostromedial tegmental nucleus to the striatum in the dog brain. Neuroscience and Behavioral Physiology, 44(6), 614–618. 10.1007/s11055-014-9959-5

[b57] Graham, K. L., Johnson, P. J., Barry, E. F., Orrico Pérez, M., Soligo, D. J., Lawlor, M., & White, A. (2021). Diffusion tensor imaging of the visual pathway in dogs with primary angle-closure glaucoma. Veterinary Ophthalmology, 24(S1), 63–74. 10.1111/vop.12824 32990378

[b58] Gunning-Dixon, F. M., Brickman, A. M., Cheng, J. C., & Alexopoulos, G. S. (2009). Aging of cerebral white matter: A review of MRI findings. International Journal of Geriatric Psychiatry, 24(2), 109–117. 10.1002/gps.2087 18637641 PMC2631089

[b59] Gurda, B. L., & Vite, C. H. (2019). Large animal models contribute to the development of therapies for central and peripheral nervous system dysfunction in patients with lysosomal storage diseases. Human Molecular Genetics, 28(R1), R119–R131. 10.1093/hmg/ddz127 31384936

[b60] Hakulinen, U., Brander, A., Ryymin, P., Öhman, J., Soimakallio, S., Helminen, M., Dastidar, P., & Eskola, H. (2012). Repeatability and variation of region-of-interest methods using quantitative diffusion tensor MR imaging of the brain. BMC Medical Imaging, 12(1), 30. 10.1186/1471-2342-12-30 23057584 PMC3533516

[b61] Hamamoto, Y., Hasegawa, D., Mizoguchi, S., Yu, Y., Wada, M., Kuwabara, T., Fujiwara-Igarashi, A., & Fujita, M. (2017). Changes in the interictal and early postictal diffusion and perfusion magnetic resonance parameters in familial spontaneous epileptic cats. Epilepsy Research, 133, 76–82. 10.1016/j.eplepsyres.2017.04.015 28458103

[b62] Harsan, L. A., Paul, D., Schnell, S., Kreher, B. W., Hennig, J., Staiger, J. F., & Elverfeldt Von, D. (2010). In vivo diffusion tensor magnetic resonance imaging and fiber tracking of the mouse brain. NMR in Biomedicine, 23(7), 884–896. 10.1002/nbm.1496 20213629

[b63] Head, E. (2013). A canine model of human aging and Alzheimer’s disease. Biochimica et Biophysica Acta—Molecular Basis of Disease, 1832(9), 1384–1389. 10.1016/j.bbadis.2013.03.016 PMC3937962 23528711

[b64] Hecht, E. E., Gutman, D. A., Bradley, B. A., Preuss, T. M., & Stout, D. (2015). Virtual dissection and comparative connectivity of the superior longitudinal fasciculus in chimpanzees and humans. NeuroImage, 108, 124–137. 10.1016/j.neuroimage.2014.12.039 25534109 PMC4324003

[b65] Henderson, F., Abdullah, K. G., Verma, R., & Brem, S. (2020). Tractography and the connectome in neurosurgical treatment of gliomas: The premise, the progress, and the potential. Neurosurgical Focus, 48(2), E6. 10.3171/2019.11.FOCUS19785 PMC7831974 32006950

[b66] Hicks, J., Platt, S., Kent, M., & Haley, A. (2017). Canine brain tumours: A model for the human disease? Veterinary and Comparative Oncology, 15(1), 252–272. 10.1111/vco.12152 25988678

[b67] Horschler, D. J., & MacLean, E. L. (2019). Leveraging brain–body scaling relationships for comparative studies. Animal Cognition, 22(6), 1197–1202. 10.1007/s10071-019-01316-8 31605247

[b68] Hubbard, M. E., Arnold, S., Zahid Bin, A., McPheeters, M., O’Sullivan Gerard, M., Tabaran, A. F., Hunt, M. A., & Pluhar, G. E. (2018). Naturally occurring canine glioma as a model for novel therapeutics. Cancer Investigation, 36(8), 415–423. 10.1080/07357907.2018.1514622 30234401

[b69] Hugenschmidt, C. E., Peiffer, A. M., Kraft, R. A., Casanova, R., Deibler, A. R., Burdette, J. H., Maldjian, J. A., & Laurienti, P. J. (2008). Relating imaging indices of white matter integrity and volume in healthy older adults. Cerebral Cortex, 18(2), 433–442. 10.1093/cercor/bhm080 17575289

[b70] Hülsmeyer, V. I., Fischer, A., Mandigers, P. J. J., DeRisio, L., Berendt, M., Rusbridge, C., Bhatti, S. F. M., Pakozdy, A., Patterson, E. E., Platt, S., Packer, R. M. A., & Volk, H. A. (2015). International Veterinary Epilepsy Task Force’s current understanding of idiopathic epilepsy of genetic or suspected genetic origin in purebred dogs. BMC Veterinary Research, 11(1), 175. 10.1186/s12917-015-0463-0 26316206 PMC4552344

[b71] Jacqmot, O., Van Thielen, B., Fierens, Y., Hammond, M., Willekens, I., Schuerbeek, P. Van, Verhelle, F., Goossens, P., Ridder De, F., Clarys, J. P., Vanbinst, A., & Mey De, J. (2013). Diffusion tensor imaging of white matter tracts in the dog brain. Anatomical Record, 296(2), 340–349. 10.1002/ar.22638 23355519

[b72] Jacqmot, O., Van Thielen, B., Michotte, A., Willekens, I., Verhelle, F., Goossens, P., Ridder De, F., Clarys, J. P., Vanbinst, A., Peleman, C., & Mey de, J. (2017). Comparison of several white matter tracts in feline and canine brain by using magnetic resonance diffusion tensor imaging. Anatomical Record, 300(7), 1270–1289. 10.1002/ar.23579 28214332

[b73] Jendrny, P., Twele, F., Meller, S., Osterhaus, A. D. M. E., Schalke, E., & Volk, H. A. (2021). Canine olfactory detection and its relevance to medical detection. BMC Infectious Diseases, 21(1), 838. 10.1186/s12879-021-06523-8 34412582 PMC8375464

[b74] Jiang, Y., & Johnson, G. A. (2011). Microscopic diffusion tensor atlas of the mouse brain. NeuroImage, 56(3), 1235–1243. 10.1016/J.NEUROIMAGE.2011.03.031 21419226 PMC3085633

[b75] Johnson, P. J., Barry, E. F., Luh, W. M., & Davies, E. (2019). The use of diffusion tractography to characterize a corpus callosum malformation in a dog. Journal of Veterinary Internal Medicine, 33(2), 743–750. 10.1111/jvim.15392 30588678 PMC6430883

[b76] Johnson, P. J., Janvier, V., Luh, W. M., FitzMaurice, M., Southard, T., & Barry, E. F. (2019). Equine stereotaxtic population average brain atlas with neuroanatomic correlation. Frontiers in Neuroanatomy, 13, 1–13. 10.3389/fnana.2019.00089 31636547 PMC6787676

[b77] Johnson, P. J., Luh, W. M., Rivard, B. C., Graham, K. L., White, A., Fitz-Maurice, M., Loftus, J. P., & Barry, E. F. (2020). Stereotactic cortical atlas of the domestic canine brain. Scientific Reports, 10(1), 1–16. 10.1038/s41598-020-61665-0 32179861 PMC7076022

[b78] Johnson, P. J., Miller, A. D., Cheetham, J., Demeter, E. A., Luh, W. M., Loftus, J. P., Stephan, S. L., Dewey, C. W., & Barry, E. F. (2021). In vivo detection of microstructural spinal cord lesions in dogs with degenerative myelopathy using diffusion tensor imaging. Journal of Veterinary Internal Medicine, 35(1), 352–362. 10.1111/jvim.16014 33350517 PMC7848345

[b79] Johnson, P. J., Pascalau, R., Luh, W. M., Raj, A., Cerda-Gonzalez, S., & Barry, E. F. (2020). Stereotaxic diffusion tensor imaging white matter atlas for the in vivo domestic feline brain. Frontiers in Neuroanatomy, 14, 1–13. 10.3389/fnana.2020.00001 32116572 PMC7026623

[b80] Kellner, E., Dhital, B., Kiselev, V. G., & Reisert, M. (2016). Gibbs-ringing artifact removal based on local subvoxel-shifts. Magnetic Resonance in Medicine, 76(5), 1574–1581. 10.1002/mrm.26054 26745823

[b81] Kowalska, D. M. (2000). Cognitive functions of the temporal lobe in the dog: A review. Progress in Neuro-Psychopharmacology and Biological Psychiatry, 24(5), 855–880. 10.1016/S0278-5846(00)00110-X 11191717

[b82] Le Bihan, D. (2003). Looking into the functional architecture of the brain with diffusion MRI. Nature Reviews Neuroscience, 4(6), 469–480. 10.1038/nrn1119 12778119

[b83] Lebel, C., Caverhill-Godkewitsch, S., & Beaulieu, C. (2010). Age-related regional variations of the corpus callosum identified by diffusion tensor tractography. NeuroImage, 52(1), 20–31. 10.1016/j.neuroimage.2010.03.072 20362683

[b84] Lebel, C., & Deoni, S. (2018). The development of brain white matter microstructure. NeuroImage, 182, 207–218. 10.1016/j.neuroimage.2017.12.097 29305910 PMC6030512

[b85] Lebel, C., Gee, M., Camicioli, R., Wieler, M., Martin, W., & Beaulieu, C. (2012). Diffusion tensor imaging of white matter tract evolution over the lifespan. NeuroImage, 60(1), 340–352. 10.1016/j.neuroimage.2011.11.094 22178809

[b86] Leblanc, A. K., Mazcko, C., Brown, D. E., Koehler, J. W., Miller, A. D., Miller, C. R., Bentley, R. T., Packer, R. A., Breen, M., Boudreau, C. E., Levine, J. M., Simpson, R. M., Halsey, C., Kisseberth, W., Rossmeisl, J. H., Dickinson, P. J., Fan, T. M., Corps, K., Aldape, K., … Gilbert, R M. (2016). Creation of an NCI comparative brain tumor consortium: Informing the translation of new knowledge from canine to human brain tumor patients. Neuro-Oncology, 18(9), 1209–1218. 10.1093/neuonc/now051 27179361 PMC4999002

[b87] Liu, C., Ye, F. Q., Newman, J. D., Szczupak, D., Tian, X., Yen, C. C.-C., Majka, P., Glen, D., Rosa, M. G. P., Leopold, D. A., & Silva, A. C. (2020). A resource for the detailed 3D mapping of white matter pathways in the marmoset brain. Nature Neuroscience, 23(2), 271–280. 10.1038/s41593-019-0575-0 31932765 PMC7007400

[b88] Löscher, W. (2022). Dogs as a natural animal model of epilepsy. Frontiers in Veterinary Science, 9, 928009. 10.3389/fvets.2022.928009 35812852 PMC9257283

[b89] Manns, J. R., & Eichenbaum, H. (2006). Evolution of declarative memory. Hippocampus, 16(9), 795–808. 10.1002/hipo.20205 16881079

[b90] Miller, A. D., Miller, C. R., & Rossmeisl, J. H. (2019). Canine primary intracranial cancer: A clinicopathologic and comparative review of glioma, meningioma, and choroid plexus tumors. Frontiers in Oncology, 9, 1151. 10.3389/fonc.2019.01151 31788444 PMC6856054

[b91] Miller, K. L., McNab, J. A., Jbabdi, S., & Douaud, G. (2012). Diffusion tractography of post-mortem human brains: Optimization and comparison of spin echo and steady-state free precession techniques. NeuroImage, 59(3), 2284–2297. 10.1016/j.neuroimage.2011.09.054 22008372 PMC3314951

[b92] Mori, S., Oishi, K., & Faria, A. V. (2009). White matter atlases based on diffusion tensor imaging. Current Opinion in Neurology, 22(4), 362–369. 10.1097/WCO.0b013e32832d954b 19571751 PMC2883814

[b93] Mori, S., Oishi, K., Jiang, H., Jiang, L., Li, X., Akhter, K., Hua, K., Faria, A. V., Mahmood, A., Woods, R., Toga, A. W., Pike, G. B., Neto, P. R., Evans, A., Zhang, J., Huang, H., Miller, M. I., van Zijl, P., & Mazziotta, J. (2008). Stereotaxic white matter atlas based on diffusion tensor imaging in an ICBM template. NeuroImage, 40(2), 570–582. 10.1016/j.neuroimage.2007.12.035 18255316 PMC2478641

[b94] Mori, S., van Zijl, P. C., Oishi, K., & Faria, A. V. (2011). MRI atlas of human white matter (2nd ed.). Elsevier. https://shop.elsevier.com/books/mri-atlas-of-human-white-matter/oishi/978-0-12-382081-5

[b95] Mori, S., & Zhang, J. (2006). Principles of diffusion tensor imaging and its applications to basic neuroscience research. Neuron, 51(5), 527–539. 10.1016/j.neuron.2006.08.012 16950152

[b96] Mukherjee, P., & McKinstry, R. C. (2006). Diffusion tensor imaging and tractography of human brain development. Neuroimaging Clinics of North America, 16(1), 19–43. 10.1016/j.nic.2005.11.004 16543084

[b97] Muratoff, V. (1893). Secundäre Degenerationen nach Zerstörung der motorischen Sphäre des Gehirns. Archives of Anatomy and Physiology (Physiol Abt), 18.

[b98] Nair, G., Carew, J. D., Usher, S., Lu, D., Hu, X. P., & Benatar, M. (2010). Diffusion tensor imaging reveals regional differences in the cervical spinal cord in amyotrophic lateral sclerosis. NeuroImage, 53(2), 576–583. 10.1016/j.neuroimage.2010.06.060 20600964

[b99] Nardone, R., Höller, Y., Taylor, A. C., Lochner, P., Tezzon, F., Golaszewski, S., Brigo, F., & Trinka, E. (2016). Canine degenerative myelopathy: A model of human amyotrophic lateral sclerosis. Zoology, 119(1), 64–73. 10.1016/j.zool.2015.09.003 26432396

[b100] Nghiem, P. P., & Kornegay, J. N. (2019). Gene therapies in canine models for Duchenne muscular dystrophy. Human Genetics, 138(5), 483–489. 10.1007/s00439-019-01976-z 30734120

[b101] Nitzsche, B., Boltze, J., Ludewig, E., Flegel, T., Schmidt, M. J., Seeger, J., Barthel, H., Brooks, O. W., Gounis, M. J., Stoffel, M. H., & Schulze, S. (2019). A stereotaxic breed-averaged, symmetric T2w canine brain atlas including detailed morphological and volumetrical data sets. NeuroImage, 187, 93–103. 10.1016/j.neuroimage.2018.01.066 29407456

[b102] Nitzsche, B., Frey, S., Collins, L. D., Seeger, J., Lobsien, D., Dreyer, A., Kirsten, H., Stoffel, M. H., Fonov, V. S., & Boltze, J. (2015). A stereotaxic, population-averaged T1w ovine brain atlas including cerebral morphology and tissue volumes. Frontiers in Neuroanatomy, 9, 1–14. 10.3389/fnana.2015.00069 26089780 PMC4455244

[b103] Oishi, K., Chang, L., & Huang, H. (2019). Baby brain atlases. NeuroImage, 185, 865–880. 10.1016/j.neuroimage.2018.04.003 29625234 PMC6170732

[b104] Oishi, K., Huang, H., Yoshioka, T., Ying, S. H., Zee, D. S., Zilles, K., Amunts, K., Woods, R., Toga, A. W., Pike, G. B., Rosa-Neto, P., Evans, A. C., Van Zijl, P. C. M., Mazziotta, J. C., & Mori, S. (2011). Superficially located white matter structures commonly seen in the human and the macaque brain with diffusion tensor imaging. Brain Connectivity, 1(1), 37–47. 10.1089/brain.2011.0005 22432953 PMC3569096

[b105] Oishi, K., Zilles, K., Amunts, K., Faria, A., Jiang, H., Li, X., Akhter, K., Hua, K., Woods, R., Toga, A. W., Pike, G. B., Rosa-Neto, P., Evans, A., Zhang, J., Huang, H., Miller, M. I., van Zijl, P. C. M., Mazziotta, J., & Mori, S. (2008). Human brain white matter atlas: Identification and assignment of common anatomical structures in superficial white matter. NeuroImage, 43(3), 447–457. 10.1016/j.neuroimage.2008.07.009 18692144 PMC2586008

[b106] O’Sullivan, M., Jones, D. K., Summers, P. E., Morris, R. G., Williams, S. C. R., & Markus, H. S. (2001). Evidence for cortical “disconnection” as a mechanism of age-related cognitive decline. Neurology, 57(4), 632–638. 10.1212/WNL.57.4.632 11524471

[b107] Ota, Y., & Shah, G. (2022). Imaging of normal brain aging. Neuroimaging Clinics of North America, 32(3), 683–698. 10.1016/j.nic.2022.04.010 35843669

[b108] Pascalau, R., Aldea, C. C., Padurean, V. A., & Szabo, B. (2016). Comparative study of the major white matter tracts anatomy in equine, feline and canine brains by use of the fibre dissection technique. Journal of Veterinary Medicine Series C: Anatomia Histologia Embryologia, 45(5), 373–385. 10.1111/ahe.12208 26394884

[b109] Petersen-Jones, S. M., & Komáromy, A. M. (2015). Dog models for blinding inherited retinal dystrophies. Human Gene Therapy Clinical Development, 26(1), 15–26. 10.1089/humc.2014.155 25671556 PMC4442585

[b110] Pieri, V., Trovatelli, M., Cadioli, M., Zani, D. D., Brizzola, S., Ravasio, G., Acocella, F., Giancamillo Di, M., Malfassi, L., Dolera, M., Riva, M., Bello, L., Falini, A., & Castellano, A. (2019). In vivo diffusion tensor magnetic resonance tractography of the sheep brain: An atlas of the ovine white matter fiber bundles. Frontiers in Veterinary Science, 6, 345. 10.3389/fvets.2019.00345 31681805 PMC6805705

[b111] Probst, M. (1901). Ueber den Bau des vollständig balkenlosen Gross-hirnes sowie über Mikrogyrie und Heterotopie der grauen Substanz. Archiv Für Psychiatrie Und Nervenkrankheiten, 34(3), 709–786. 10.1007/BF02680175

[b112] Qiu, D., Tan, L. H., Zhou, K., & Khong, P. L. (2008). Diffusion tensor imaging of normal white matter maturation from late childhood to young adulthood: Voxel-wise evaluation of mean diffusivity, fractional anisotropy, radial and axial diffusivities, and correlation with reading development. NeuroImage, 41(2), 223–232. 10.1016/j.neuroimage.2008.02.023 18395471

[b113] Rakic, P., & Yakovlev, P. I. (1968). Development of the corpus callosum and cavum septi in man. The Journal of Comparative Neurology, 132(1), 45–72, 10.1002/cne.901320103 5293999

[b114] Sakai, S. T., Stanton, G. B., & Isaacson, L. G. (1993). Thalamic afferents of area 4 and 6 in the dog: A multiple retrograde fluorescent dye study. Anatomy and Embryology, 188(6), 551–559. 10.1007/BF00187010 8129177

[b115] Schachter, S., & Singer, J. (1962). Cognitive, social, and physiological determinants of emotional state. Psychological Review, 69(5), 379–399. 10.1037/h0046234 14497895

[b116] Singer, M. (1962). The Brain of the Dog in Section © 1962 Marcus Singer, renewed 1990. From BrainMaps.org (http://brainmaps.org/).

[b117] Sivakanthan, S., Neal, E., Murtagh, R., & Vale, F. L. (2016). The evolving utility of diffusion tensor tractography in the surgical management of temporal lobe epilepsy: A review. Acta Neurochirurgica, 158(11), 2185–2193. 10.1007/s00701-016-2910-5 27566714

[b118] Smith, S. M., Jenkinson, M., Woolrich, M. W., Beckmann, C. F., Behrens, T. E. J., Johansen-Berg, H., Bannister, P. R., De Luca, M., Drobnjak, I., Flitney, D. E., Niazy, R. K., Saunders, J., Vickers, J., Zhang, Y., De Stefano, N., Brady, J. M., & Matthews, P. M. (2004). Advances in functional and structural MR image analysis and implementation as FSL. NeuroImage, 23(Suppl. 1), S208–S219. 10.1016/j.neuroimage.2004.07.051 15501092

[b119] Stolzberg, D., Wong, C., Butler, B. E., & Lomber, S. G. (2017). Catlas: An magnetic resonance imaging-based three-dimensional cortical atlas and tissue probability maps for the domestic cat (*Felis catus*). Journal of Comparative Neurology, 525(15), 3190–3206. 10.1002/cne.24271 28653335

[b120] Story, B. D., Miller, M. E., Bradbury, A. M., Million, E. D., Duan, D., Taghian, T., Faissler, D., Fernau, D., Beecy, S. J., & Gray-Edwards, H. L. (2020). Canine models of inherited musculoskeletal and neurodegenerative diseases. Frontiers in Veterinary Science, 7, 80. 10.3389/fvets.2020.00080 32219101 PMC7078110

[b121] Sztriha, L. (2005). Spectrum of corpus callosum agenesis. Pediatric Neurology, 32(2), 94–101. 10.1016/j.pediatrneurol.2004.09.007 15664768

[b122] Talwar, P., Kushwaha, S., Chaturvedi, M., & Mahajan, V. (2021). Systematic review of different neuroimaging correlates in mild cognitive impairment and Alzheimer’s disease. Clinical Neuroradiology, 31(4), 953–967. 10.1007/s00062-021-01057-7 34297137

[b123] Tournier, J. D., Smith, R., Raffelt, D., Tabbara, R., Dhollander, T., Pietsch, M., Christiaens, D., Jeurissen, B., Yeh, C. H., & Connelly, A. (2019). MRtrix3: A fast, flexible and open software framework for medical image processing and visualisation. NeuroImage, 202, 116137. 10.1016/j.neuroimage.2019.116137 31473352

[b124] Urkasemsin, G., & Olby, N. J. (2014). Canine paroxysmal movement disorders. Veterinary Clinics of North America—Small Animal Practice, 44(6), 1091–1102. 10.1016/j.cvsm.2014.07.006 25441627

[b125] Veraart, J., Novikov, D. S., Christiaens, D., Ades-aron, B., Sijbers, J., & Fieremans, E. (2016). Denoising of diffusion MRI using random matrix theory. NeuroImage, 142, 394–406. 10.1016/j.neuroimage.2016.08.016 27523449 PMC5159209

[b126] Vite, C. H., & Head, E. (2014). Aging in the canine and feline brain. Veterinary Clinics of North America—Small Animal Practice, 44(6), 1113–1129. 10.1016/j.cvsm.2014.07.008 25441628 PMC4254595

[b127] Vogt, B. A. (2019). Cingulate cortex in the three limbic subsystems. Handbook of Clinical Neurology, 166, 39–51. 10.1016/B978-0-444-64196-0.00003-0 31731924

[b128] Wang, R., Benner, T., Sorensen, A. G., & Wedeen, V. J. (2007). Diffusion toolkit: A software package for diffusion imaging data processing and tractography. Proceedings of the International Society for Magnetic Resonance in Medicine, 15, 3720. http://cds.ismrm.org/ismrm-2007/files/03720.pdf

[b129] Wang-Leandro, A., Dennler, M., & Beckmann, K. M. (2018). Presence of probst bundles indicate white matter remodeling in a dog with corpus callosum hypoplasia and dysplasia. Frontiers in Veterinary Science, 5, 260. 10.3389/FVETS.2018.00260 30406119 PMC6204354

[b130] Wu, Y., Zhang, F., Makris, N., Ning, Y., Norton, I., She, S., Peng, H., Rathi, Y., Feng, Y., Wu, H., & O’Donnell, L. J. (2018). Investigation into local white matter abnormality in emotional processing and sensorimotor areas using an automatically annotated fiber clustering in major depressive disorder. NeuroImage, 181, 16–29. 10.1016/j.neuroimage.2018.06.019 29890329 PMC6415925

[b131] Yeon Kim, S., Ik Kim Seung-Koo Lee, D., Mori, S., Kim Joon, D., & Young, S. (2004). Callosal dysgenesis altered hemispheric fiber connection in diffusion tensor MR imaging visualizes the. American Journal of Neuroradiology, 25(1). http://www.ajnr.org/content/25/1/PMC7974170 14729523

[b132] Yogarajah, M., & Duncan, J. S. (2008). Diffusion-based magnetic resonance imaging and tractography in epilepsy. Epilepsia, 49(2), 189–200. 10.1111/j.1528-1167.2007.01378.x 17941849

[b133] Yon, M., Bao, Q., Chitrit, O. J., Henriques, R. N., Shemesh, N., & Frydman, L. (2020). High-resolution 3D in vivo brain diffusion tensor imaging at ultrahigh fields: Following maturation on juvenile and adult mice. Frontiers in Neuroscience, 14, 590900. 10.3389/fnins.2020.590900 33328861 PMC7714913

[b134] Yushkevich, P. A., Piven, J., Hazlett, H. C., Smith, R. G., Ho, S., Gee, J. C., & Gerig, G. (2006). User-guided 3D active contour segmentation of anatomical structures: Significantly improved efficiency and reliability. NeuroImage, 31(3), 1116–1128. 10.1016/j.neuroimage.2006.01.015 16545965

[b135] Zakszewski, E., Adluru, N., Tromp, D. P. M., Kalin, N., & Alexander, A. L. (2014). A diffusion-tensor-based white matter atlas for rhesus macaques. PLoS One, 9(9), e107398. 10.1371/journal.pone.0107398 25203614 PMC4159318

[b136] Zhang, F., Wu, Y., Norton, I., Rigolo, L., Rathi, Y., Makris, N., & O’Donnell, L. J. (2018). An anatomically curated fiber clustering white matter atlas for consistent white matter tract parcellation across the lifespan. NeuroImage, 179, 429–447. 10.1016/j.neuroimage.2018.06.027 29920375 PMC6080311

[b137] Zhang, Y., Zhang, J., Oishi, K., Faria, A. V., Jiang, H., Li, X., Akhter, K., Rosa-Neto, P., Pike, G. B., Evans, A., Toga, A. W., Woods, R., Mazziotta, J. C., Miller, M. I., van Zijl, P. C. M., & Mori, S. (2010). Atlas-guided tract reconstruction for automated and comprehensive examination of the white matter anatomy. NeuroImage, 52(4), 1289–1301. 10.1016/j.neuroimage.2010.05.049 20570617 PMC2910162

