# Supplementary material for: A diffusion tensor imaging white matter atlas of the domestic canine brain
Source: Imaging Neurosci (Camb). 2024 Aug 30;2:imag-2-00276. doi: 10.1162/imag_a_00276 (PMC11409835; doi:10.1162/imag_a_00276)
Supplement: Supplementary Material [file imag_a_00276-supp.pdf]

Deterministic White Matter Dissection Guide For The Canine White Matter Atlas.

Tractography dissections of the population average diffusion dataset tractogram was performed using TrackVis software. The major projection, association, commissural and cerebellar fasciculi were dissected using both human and animal anatomic references. Dissections were performed using seed regions of interest. These ROIs were then used to include or exclude white matter passing through that region. Region of interest placement was performed in reference to published anatomic atlases that document the lobar regions of the brain and other structures. The figures below demonstrate the placement of ROIs or specific regions of the brain to allow for dissection of specific fasciculi. In some cases large regions of interest were placed to form exclusion zones, allowing differentiation of closely associated shorter and longer white matter pathways, such as the thalamic radiation vs the corticospinal tract.

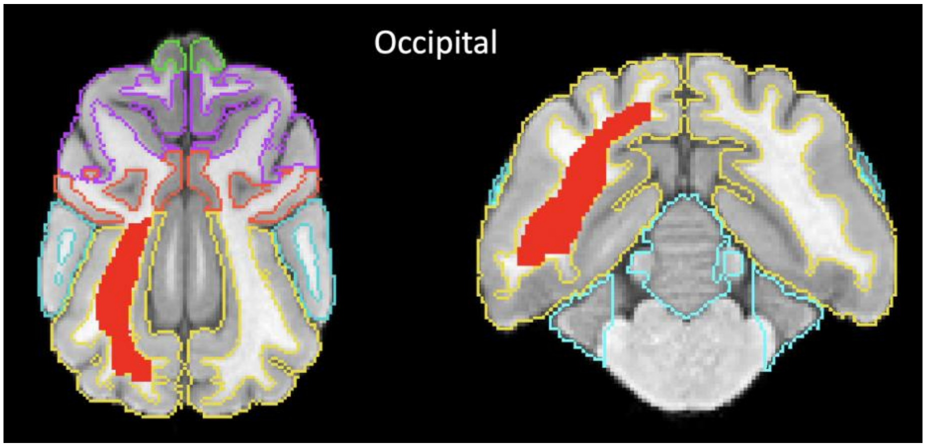

Occipital ROIs were placed within the central white matter of the occipital lobe in both dorsal and transverse planes. These ROIs were utilized for dissections of multiple different fasciculi including the optic radiation, olfactory occipital tract and inferior and superior fronto-occipital tracts (yellow boundary depicts the margins of the occipital lobe in the dog).

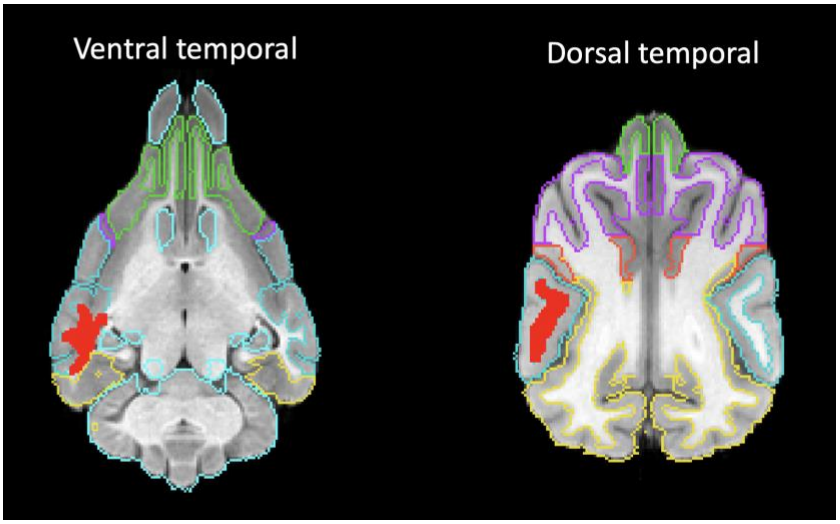

Temporal ROIs were placed within the white matter either ventrally or dorsally. These ROIs were utilized for dissecting tracts such as the uncinate and superficial longitudinal fasciculus (cyan boundary depicts the temporal lobe).

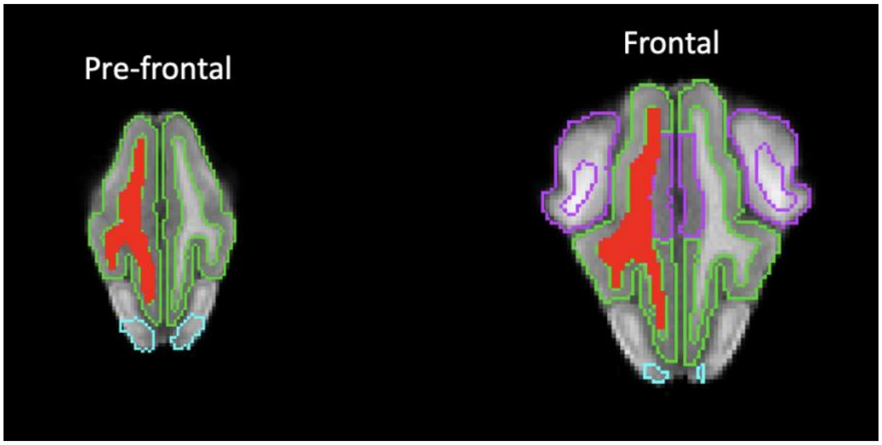

Frontal and prefrontal ROIs were placed within the white matter of the frontal lobe (green outline). This was used for dissection of white matter pathways such as the fronto-occipital fasciculi and uncinate fasciculi.

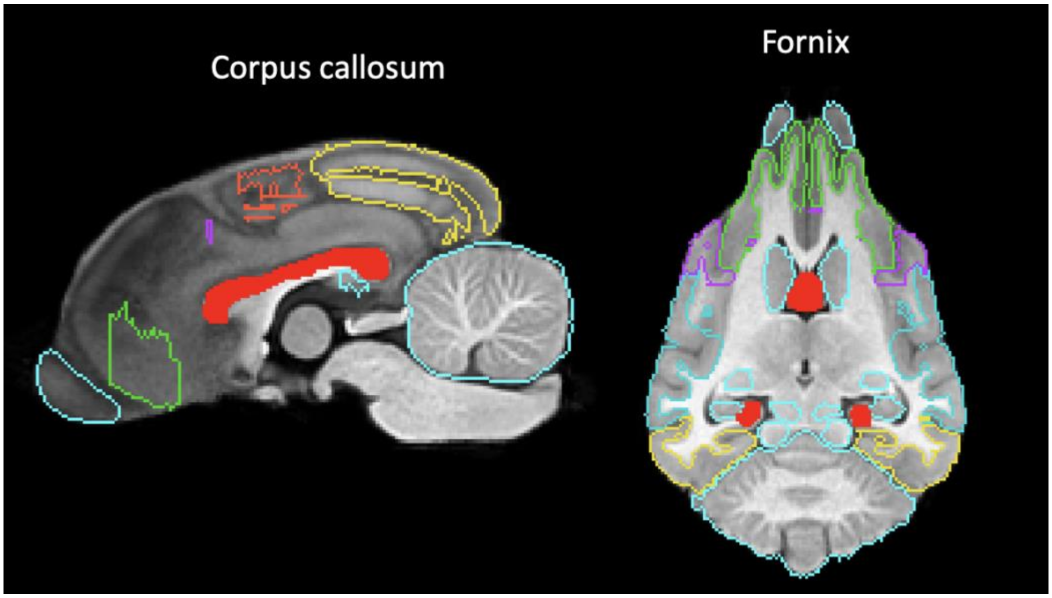

The corpus callosum was dissected using a midline ROI as depicted above and described previously (Johnson, Barry, et al., 2019). The fornix ROIs were placed on the body and crura of the fornix as depicted.

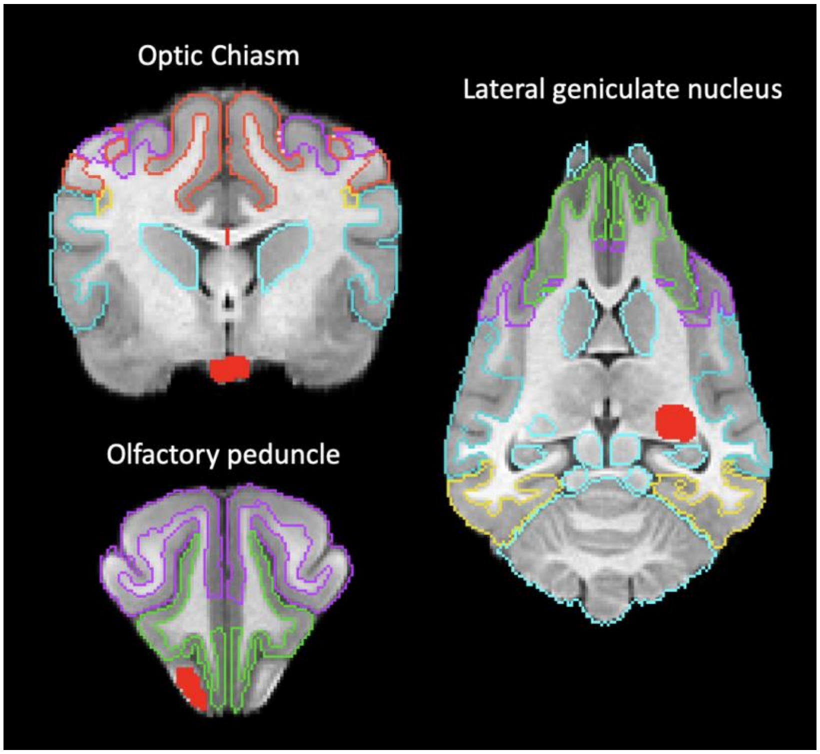

Optic chiasm and lateral geniculate nuclei ROIs were used for dissection of the optic pathway as previously described (Andrews et al., 2021). The olfactory white matter was seeded from ROIs placed in the olfactory peduncle (Andrews et al., 2022).

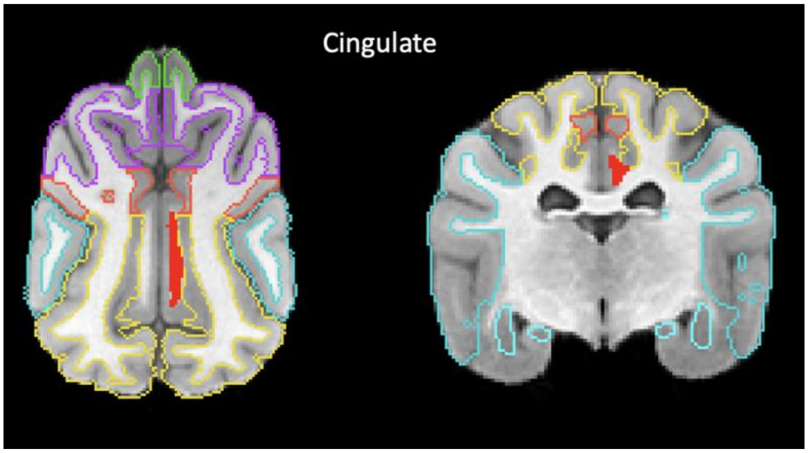

Cingulate ROIs were generated to dissect the cingulate white matter pathway.

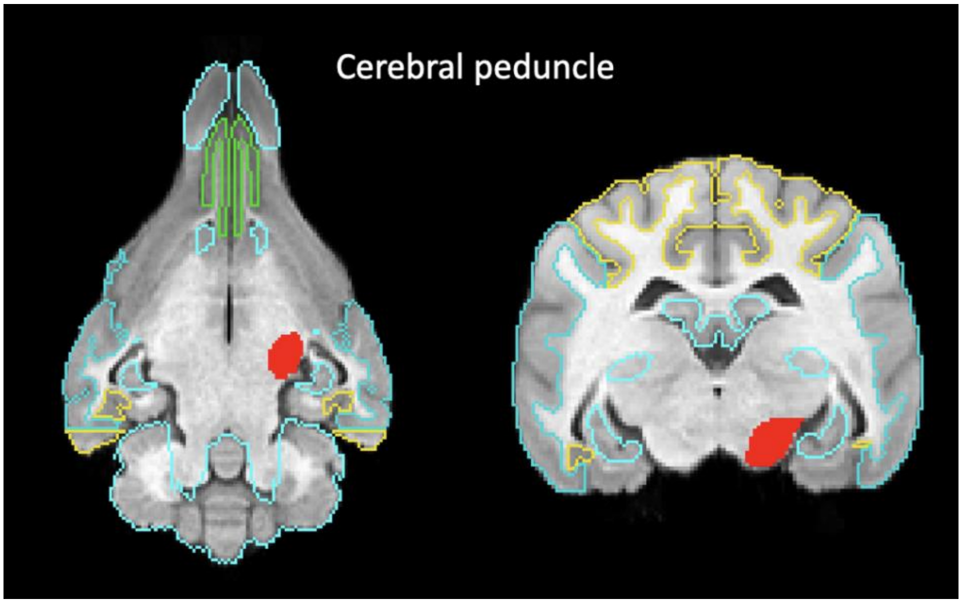

Cerebral peduncle ROIs were generated to dissect the corticospinal tract
